# Mutations in apoptosis-inducing factor cause X-linked recessive auditory neuropathy spectrum disorder

**DOI:** 10.1136/jmedgenet-2014-102961

**Published:** 2015-05-18

**Authors:** Liang Zong, Jing Guan, Megan Ealy, Qiujing Zhang, Dayong Wang, Hongyang Wang, Yali Zhao, Zhirong Shen, Colleen A Campbell, Fengchao Wang, Ju Yang, Wei Sun, Lan Lan, Dalian Ding, Linyi Xie, Yue Qi, Xin Lou, Xusheng Huang, Qiang Shi, Suhua Chang, Wenping Xiong, Zifang Yin, Ning Yu, Hui Zhao, Jun Wang, Jing Wang, Richard J Salvi, Christine Petit, Richard J H Smith, Qiuju Wang

**Affiliations:** 1 Department of Otolaryngology-Head and Neck Surgery, Institute of Otolaryngology, PLA General Hospital, Beijing, China; 2 Molecular Otolaryngology and Renal Research Laboratories and the Department of Otolaryngology-Head and Neck Surgery, University of Iowa, Iowa City, Iowa, USA; 3 Department of Otolaryngology-Head & Neck Surgery, Stanford University School of Medicine, Stanford, California, USA; 4 Beijing Institute of Otorhinolaryngology, Beijing Tongren Hospital, Capital Medical University, Beijing, China; 5 National Institute of Biological Sciences, Beijing, China; 6 Department of Communicative Disorders & Sciences, Center for Hearing and Deafness, University at Buffalo, Buffalo, New York, USA; 7 Department of Radiology, PLA General Hospital, Beijing, China; 8 Department of Neurology, PLA General Hospital, Beijing, China; 9 Key Laboratory of Mental Health, Institute of Psychology, Chinese Academy of Sciences, Beijing, China; 10 BGI-Shenzhen, Shenzhen, China; 11 Unité de Génétique et Physiologie de l'Audition, Institut Pasteur, Collège de France, Paris, France

**Keywords:** Clinical genetics, Genetic heterogeneity, Mutation, Auditory neuropathy spectrum disorder

## Abstract

**Background:**

Auditory neuropathy spectrum disorder (ANSD) is a form of hearing loss in which auditory signal transmission from the inner ear to the auditory nerve and brain stem is distorted, giving rise to speech perception difficulties beyond that expected for the observed degree of hearing loss. For many cases of ANSD, the underlying molecular pathology and the site of lesion remain unclear. The X-linked form of the condition, AUNX1, has been mapped to Xq23-q27.3, although the causative gene has yet to be identified.

**Methods:**

We performed whole-exome sequencing on DNA samples from the AUNX1 family and another small phenotypically similar but unrelated ANSD family.

**Results:**

We identified two missense mutations in *AIFM1* in these families: c.1352G>A (p.R451Q) in the AUNX1 family and c.1030C>T (p.L344F) in the second ANSD family. Mutation screening in a large cohort of 3 additional unrelated families and 93 sporadic cases with ANSD identified 9 more missense mutations in *AIFM1*. Bioinformatics analysis and expression studies support this gene as being causative of ANSD.

**Conclusions:**

Variants in *AIFM1* gene are a common cause of familial and sporadic ANSD and provide insight into the expanded spectrum of *AIFM1*-associated diseases. The finding of cochlear nerve hypoplasia in some patients was *AIFM1*-related ANSD implies that MRI may be of value in localising the site of lesion and suggests that cochlea implantation in these patients may have limited success.

## Introduction

Auditory neuropathy spectrum disorder (ANSD) is characterised by absent or severely abnormal inner hair cell (IHC) function as measured by auditory brainstem responses (ABRs), with preservation of outer hair cell (OHC) function as indicated by otoacoustic emission (OAE) and/or cochlear microphonic (CM) testing. First described by Starr *et al* in 1996,^1^ patients with ANSD present with variable degrees of unilateral or bilateral hearing impairment accompanied by poor speech discrimination and poor word understanding especially in the presence of noise. The prevalence of ANSD varies from 0.5% to 15% among hearing-impaired patients, with an incidence of about 13% in children with severe-to-profound hearing loss.^2–4^ Consistent with physiological tests of auditory function, ANSD can be caused by lesions of the IHC, IHC–auditory nerve synapse, auditory nerve or auditory cortex.^5–7^

In many cases of ANSD, the molecular pathology remains unclear, with underlying aetiologies running the gamut of genetic abnormalities, toxic/metabolic derangements, infections, immunological causes and drugs.^8^ ^9^ Forty per cent of ANSD is estimated to have a genetic basis with autosomal-dominant, autosomal-recessive, mitochondrial and X-linked inheritance all reported.^3^ The list of causative genes includes *OTOF*, *PJVK*, *DIAPH3* and *mtDNA* (*m.1095T>C*) in non-syndromic ANSD and *PMP22*, *MPZ*, *TMEM126A* and *DDDP* in syndromic ANSD, although other genetic aetiologies await discovery.^2^

In 2006, we reported a large Chinese family with X-linked progressive auditory and peripheral sensory neuropathy, and mapped this ANSD locus (AUNX1) to chrXq23-27.3.^10^ Using whole-exome sequencing (WES), we have identified the causal AUNX1 gene as *AIFM1* and show that variants in this gene are a common cause of familial and sporadic ANSD. This finding is noteworthy because *AIFM1* mutations are also associated with mitochondrial encephalomyopathy, prenatal ventriculomegaly and Cowchock syndrome, three disorders characterised by developmental disabilities, motor dysfunction, muscle weakness and brain abnormalities as resolved by MRI.^11–13^ Our work expands the spectrum of *AIFM1*-associated phenotypes and mandates screening of *AIFM1* in small pedigrees with apparent autosomal-recessive ANSD if X-linked inheritance cannot be excluded.

## Methods

### Family ascertainment and clinical evaluation

Five unrelated Chinese families (AUNX1, 7170, 0223, 2724 and 2423) and 93 sporadic male patients diagnosed with ANSD were ascertained through the Department of Otolaryngology, Head and Neck Surgery, Chinese PLA General Hospital, from November 2000 to June 2014. The phenotype of the AUNX1 family has been reported and includes auditory neuropathy and delayed peripheral sensory neuropathy inherited in an X-linked recessive pattern. The other four families had a similar clinical phenotype. In all cases, genes commonly involved in ANSD such as *OTOF*, *PJVK* and *DIAPH3* were excluded by Sanger sequencing. Acquired causes of ANSD such as prematurity, hyperbilirubinemia, anoxia, hypoxia, congenital brain abnormalities, perinatal intracranial haemorrhage, asphyxia and ototoxic drug exposure were excluded by medical history.

All participants were examined by a multidisciplinary team of healthcare providers that included a neurologist, otolaryngologist and audiologist. The evaluation consisted of a comprehensive medical history, physical examination with careful clinical assessment for peripheral neuropathies, pure-tone audiometry, tympanometry, acoustic reflex testing, ABR, distortion-product OAE testing and electrocochleography. The diagnosis of ANSD was based on recognised criteria (Guidelines Development Conference at NHS 2008, Como, Italy). Neurological examinations included assessments of cranial nerve function, motor activity, muscle weakness, sensory impairment and reflexes. Electrophysiological studies including needle electromyography (EMG), nerve conduction velocity (NCV) and somatosensory evoked potential (SEP) in seven patients from the four unrelated families were carried out. The EMG was performed in the abductor pollicis brevis, tibialis anterior and vastus lateralis muscles. Motor nerve conduction velocities (MCVs) of tibial, peroneal, median and ulnar nerves, as well as sensory nerve conduction velocities (SCVs) of sural, median and ulnar nerves, were obtained. Amplitudes of compound muscle action potential (CMAPs) and sensory nerve action potential (SNAPs) were measured from positive to negative peak values. The Mini Mental State Examination (MMSE) on the three patients (III: 1, III: 9 and III: 11) of family 2423 was conducted to assess the cognitive function. Select patients underwent MRI of brain, temporal high-resolution CT and electrocardiography. Serum enzymes related to energy metabolism, such as lactate dehydrogenase and creatine kinase, were measured.

**Figure 1:**
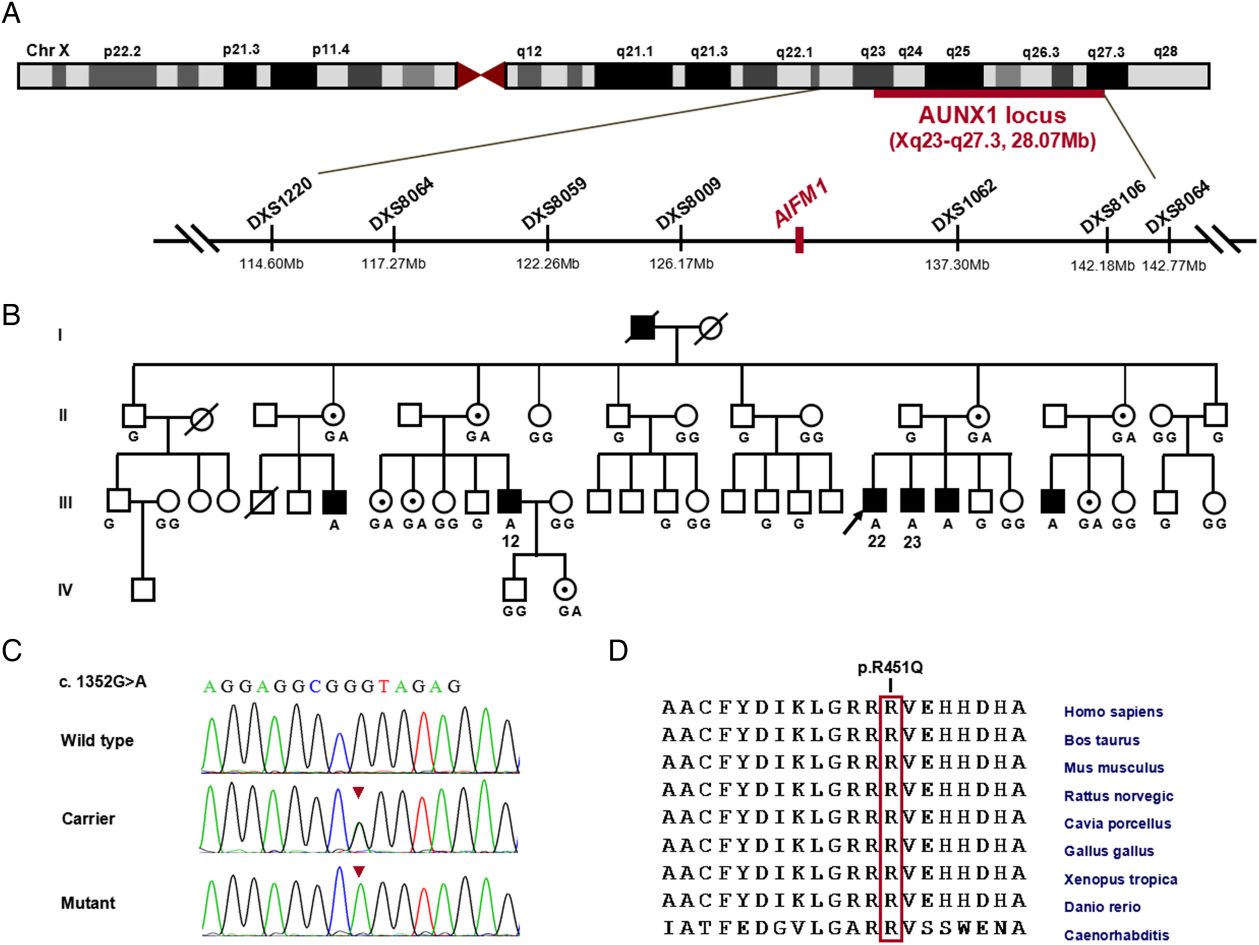
Identification of the disease-causing *AIFM1* mutation in the AUNX1 family segregating auditory and peripheral neuropathy. (A) Schematic genetic and physical map of the AUNX1 locus on chromosome Xq23-q27.3. The location of the *AIFM1* gene is indicated (Mb, million bps). (B) The phenotype in AUNX1 family co-segregates with the c.1352G>A (p.R451Q) mutation in *AIFM1*. The genotypes at c.1352 for the family members are given: G or GG means hemi- or homozygous for the wild-type (WT) sequence, GA means c.1352G>A heterozygous, and A denotes the mutation in hemizygous form. Whole-exome sequencing was completed on subject III: 12. (C) Sequence chromatograms of exon 13 of *AIFM1* show the c.1352G>A (p.R451Q) mutation (arrowhead) in affected males (hemizygote) and female carriers (heterozygote). A homozygous WT sequence is shown on the bottom. (D) Multiple sequence alignment depicts evolutionary conservation of amino acid residue Arg451 (red vertical bar) across human, bovine, mouse, chicken, Xenopus and zebrafish.

**Figure 2:**
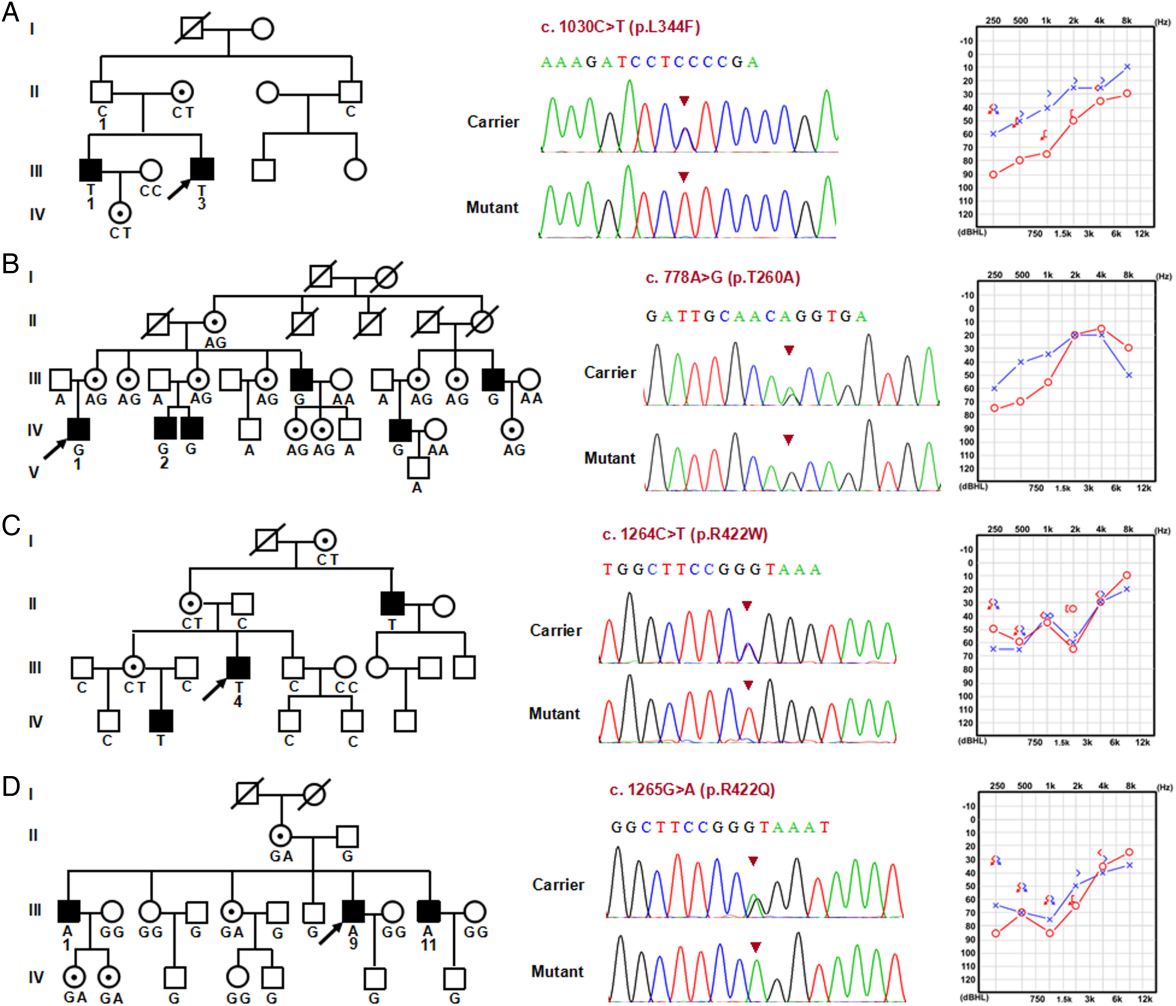
The four auditory neuropathy spectrum disorder families segregating *AIFM1* mutations. Pedigree, sequence results and typical audiogram of each family are shown. Missense mutations c.1030C>T (p.L344F), c.778A>G (p.T260A), c.1264C>T (p.R422W) and c.1265G>A (p.R422Q) were identified in family 0223 (A), 7170 (B), 2724 (C) and 2423 (D), respectively. These mutations co-segregate with auditory and peripheral sensory neuropathy while carriers have normal hearing and sensory ability. The genotypes at c.1030, c.778, c.1264 and c.1265 for the available members in the corresponding family are given respectively. Whole-exome sequencing was completed on three persons in family 0223 (II: 1, III: 1 and III: 3). Needle electromyography and nerve conduction studies were performed on the individuals from family 7170 (IV: 2), family 0223 (III: 3), family 2724 (II: 4) and family 2423 (III: 1, III: 9 and III: 11). The Mini Mental State Examination was conducted on the three patients in family 2423 (III: 1, III: 9 and III: 11).

Peripheral blood samples were obtained and genomic DNA was extracted according to standard procedures. WES was performed on one person from the AUNX1 family (III: 12) and three persons from the 0223 family (II: 1, III: 1 and III: 3). Results were confirmed and validated by Sanger sequencing in these persons and other available family members (figures 1B and 2A). Three other small families (7170, 2724 and 2423, figure 2B–D) and 93 unrelated sporadic cases with ANSD were analysed by Sanger sequencing. Five hundred ethnicity-matched individuals (250 men and 250 women) with normal hearing were recruited as normal controls.

### Whole-exome sequencing

Quantified, high-quality genomic DNA (2 µg per person) from one individual from family AUNX1 (III: 12) and three individuals from family 0223 (II: 1, III: 1 and III: 3) was used for WES. Each genomic DNA sample was captured using Agilent SureSelectXT Human All Exon V5 technology (Agilent Technologies, Santa Clara, California, USA) and enriched libraries were sequenced using the HiSeq 2000 platform (Illumina, San Diego, California, USA). Raw image files were processed on the Illumina Pipeline V.1.6 using default parameters, and sequences generated as 75–90 bp paired-end reads were aligned to NCBI37/hg19 assembly. Duplicate reads were removed using Picard (http://picard.sourceforge.net), and clean reads localised to the target region were collected and analysed by SOAPsnp (V.1.03).^14^ Local realignment of insertions and deletions (indels) and variant annotation were performed using the Genome Analysis Toolkit (http://www.broadinstitute.org/gatk/).^15^ By previously described criteria,^16^ ^17^ the low-quality variations were filtered out.

Target enrichment was analysed using NGSrich.^18^ Variants were filtered against 1000 Genomes data, and all variants with a minor allele frequency (MAF) >1% were removed from the analysis. Functional annotation of genetic variants was performed using ANNOVAR (http://www.openbioinformatics.org/annovar/). Candidate variants were Sanger validated.

### Mutation screening of *AIFM1*

*AIFM1* (NC_000023.10) contains 16 exons. Thirty-two primers (16 primer pairs) were designed using Primer V.3.0 software and synthesised by Invitrogen by Life Technology (Beijing, China) to amplify each exon and exon–intron boundaries (see online supplementary table S1). PCR was performed with PE9700 thermocyclers (Applied Biosystems) using standard conditions. Amplified products from all ANSD cases and controls were gel purified and sequenced (ABI 3730, Applied Biosystems). Nucleotide alterations were identified by sequence alignment with the NCBI Reference Sequence (RefSeq) using DNAStar software V.5.0 (DNASTAR, Madison, Wisconsin, USA).

### Evaluation of the pathogenicity

Pathogenicity was assessed using PolyPhen-2 (Polymorphism Phenotyping V.2, http://genetics.bwh.harvard.edu/pph2/index.shtml), SIFT (http://sift.jcvi.org/), Protein Variation Effect Analyzer (PROVEAN) (http://provean.jcvi.org/index.php) and Mutation accessor (http://mutationassessor.org/).

### Immunofluorescent staining in mouse inner ear

All experimental procedures were approved by the Institutional Animal Care and Use Committee of the University at Buffalo that conform to the guidelines issued by the National Institutes of Health. Adult mice (C57, 2 months of age) were used for immunofluorescence studies. The cochlear tissues of basilar membrane, spiral ligament and the vestibular end-organ of saccule macula were micro-dissected out as has been described.^19^ ^20^ After incubation at 4°C for 24 h with 1% Triton X100 and 5% goat serum in 0.1 M phosphate buffered saline (PBS) containing AIF primary antibody (rabbit monoclonal antibody against AIF, 1:100, Cat# ab32516, Abcam), the specimens were then incubated with tetramethylrhodamine isothiocyanate dextran-conjugated goat antirabbit secondary antibody (1:500, Cat# F6005, Sigma) in PBS for 2 h at room temperature. The speciments were next immersed in Alexa-488-conjugated phalloindin (1:200, Cat#A12379, Invitrogen) for 40 min to label the stereocilia and cuticular plate of the cochlear and vestibular sensory hair cells, and the F-actin in the gap-junction of marginal cells of stria vascularis. The nuclei of tissues were also labelled with 4′,6-diamidino-2-phenylindole dihydrochloride for 30 min. Immunoreactive products were observed under a confocal laser scanning microscope. As a negative control, the primary antibodies were omitted.

## Results

### Identification of missense mutations in *AIFM1* by WES

To identify the causative variant at the AUNX1 locus, we completed exome sequencing of the proband in family AUNX1. Ninety-nine variants were identified within the AUNX1 locus. After filtering out synonymous changes and variants found in <85% of reads (inconsistent with X-linked inheritance in individual III: 12, an affected man), 24 variants remained: 17 non-synonymous variants, 2 in-frame indels and 5 frameshift indels. Based on incidence data for non-syndromic hearing loss and auditory neuropathy, we excluded variants with an MAF >0.001.^21^ ^22^ Three variants remained—*AIFM1* chrX 129267384:G>A; *HS6ST2* chrX 131762528:G>A and *VCX3A* chrX 6452043:C>A—none of which are reported in the NHLBI Go Exome Sequencing Project (ESP) (6503 individuals) or the 1000 Genomes Project (1000G) (1092 individuals). Because *VCX3A* is expressed only in male germ cells, it was not considered further.^23^ ^24^ Both of the remaining variants were confirmed in the proband by Sanger sequencing, but only AIFM1 p.R451Q co-segregated with the phenotype in the extended AUNX1 family (eight informative meioses were tested; figure 1A–C). This variant was not found in a screen of 500 normal-hearing ethnicity-matched controls (250 women, 250 men, 750 X chromosomes).

To identify the causative variant in the second family (0223), we completed exome sequencing of the proband (III: 1), his affected brother (III: 3) and unaffected father (II: 1). WES generated an average of 12.3 Gb of sequence, with at least 120× average coverage for each individual as paired-end, 90 bp reads, indicating the high quality of sequencing (see online supplementary figures S1 and S2 and table S2). After mapping to the reference genome sequence, >99.0% of the targeted bases were covered sufficiently to pass quality assessment for calling single-nucleotide polymorphisms (SNPs) and short indels (see online supplementary table S2). We identified an average of ∼20 700 SNPs in coding regions (exonic), an average of 129 variants (SNPs and indels) within 2 bp of an exon/intron boundary that may affect splicing, and an average of 1270 indels in coding regions (see online supplementary table S3). Because the two affected individuals share the causal variant when compared with their normal-hearing father, a total of 807 variants were retained after filtering against SNP and Indel databases including dbSNP 141, 1000G, Hapmap 8 and YH (see online supplementary tables S4 and S5). Among them, 213 variants (including 129 non-synonymous SNPs and splice sites, as well as 84 indels) were predicted to have a functional impact (see online supplementary tables S6 and S7). Because inheritance was consistent with an autosomal or X-linked recessive pattern based on family pedigrees, candidate pathogenic variants selected for further analysis were rare homozygous or hemizygous nonsense, missense, splice site and indel variants with allele frequencies of ≤0.005 in public variant databases. Combined with the predicted effect on protein function by SIFT, PolyPhen2 and Mutation Assessor programs, we identified five variants (including one rare SNP and four indels) to be candidates (see online supplementary table S8). After Sanger sequencing and genotyping in all available family members, the only variant segregating with the phenotype (auditory and peripheral sensory neuropathy) of family 0223 was c.1030C>T (p.L344F) in the AIFM1 gene (figure 2A). This variant is recorded in dbSNP (rs184474885, http://www.ncbi.nlm.nih.gov/projects/SNP) with a very low MAF (A=0.0005, 2/3775). Interestingly, although this variant is recorded as an SNP in 1000G, it was not found in the screen of 500 normal-hearing ethnicity-matched controls in Chinese populations (mentioned above).

### Mutation spectrum of *AIFM1* in familial and sporadic ANSD

**Figure 3:**
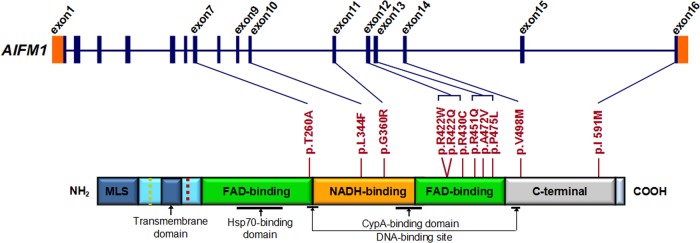
*AIFM1* mutation screening in patients with familial and sporadic auditory neuropathy spectrum disorder (ANSD) with or without peripheral neuropathy. Graphical representation of *AIFM1* structure (upper panel) and its encoded protein (lower panel). *AIFM1* gene has 16 exons. As a flavoprotein with an oxidoreductase enzymatic activity, AIFM1 contains a flavin adenine dinucleotide (FAD)-bipartite domain (in green), a reduced nicotinamide adenine dinucleotide (NADH)-binding motif (in orange) and a C-terminal domain (in grey). It also has a mitochondria localisation sequence (in blue) located in the N-terminal region (reference: http://atlasgeneticsoncology.org//Genes/AIFM1ID44053chXq25.html). The positions of 11 mutations identified in familial and sporadic ANSD cases are shown between the two diagrams (blue lines and red bars). The c.1352G>A (p.R451Q) mutation identified in the AUNX1 family is located in exon 13, which corresponds to the second FAD domain. The longest isoform of *AIFM1* (NM_004208.3; NP_004199.1) was used as the reference sequence for mutation nomenclature.

To investigate the contribution of *AIFM1* to ANSD in China, we screened this gene for mutations in our extended familial and sporadic ANSD cohort; identifying 10 more novel missense mutations in three additional families and 11 of 93 (10%) men with an ANSD phenotype (figure 3, table 1 and online supplementary table S9). In all cases of familial ANSD, the identified missense mutations (c.778A>G [p.T260A], c.1030C>T [p.L344F], c.1264C>T [p.R422W], c.1265G>A [p.R422Q], c.1352G>A [p.R451Q]) completely segregated with the auditory and peripheral sensory neuropathy phenotype, with female carriers not reporting any signs of ANSD or peripheral sensory neuropathy (figures 1B and 2). Two variants identified in familial ANSD, c.1030C>T (p.L344F) and c.1264C>T (p.R422W), were also detected in sporadic ANSD cases, with similar phenotypes (table 1). All of the 10 additional *AIFM1* mutations were also absent in the screen of normal-hearing ethnicity-matched controls (750 X chromosomes). None of these variants were found in ESP or 1000G except c.1030C>T (p.L344F) (see online supplementary table S10).

**Table 1:** Summary of the clinical phenotypes for cases with AIFM1 mutations

Cases ID	7170*	0223*	1302	1757	7187	1747	2724*	3033	6962	2423*	0077	AUNX1*	1806	0046	4678	3305
Mutation† detected	c.778A>G (p.T260A)	c.1030C>T (p.L344F)‡				c.1078G>C (p.G360R)	c.1264C>T (p.R422W)§			c.1265G>A (p.R422Q)	c.1288C>T (p.R430C)	c.1352G>A (p.R451Q)	c.1415C>T (p.A472V)	c.1424C>T (p.P475L)	c.1492G>A (p.V498M)	c.1773C>G (p.I591M)
Hearing loss degree¶	Mild	Mild	Mild	Mild	Moderate	Moderate	Moderate	Mild	Mild	Moderate	Mild	Moderate	Mild	Moderate	Moderate	Mild
Tinnitus	+	−	+	+	+	+	+	+	−	+	−	+	+	−	+	+
Vertigo	−	−	−	−	−	−	−	−	−	−	−	−	−	−	−	−
Unsteadiness	+	+	−	−	−	+	+	+	−	+	−	+	−	−	−	−
Numbness of extremities	+	+	−	+	−	+	+	+	−	+	−	+	−	−	−	−
Visual impairment	−	−	−	−	−	Myopia	−	−	−	Myopia	−	−	Myopia	−	−	Myopia
Foot deformity	−	−	−	−	−	−	−	−	−	−	−	−	−	−	−	−
Muscle atrophy	−	−	−	−	−	−	−	−	−	−	−	−	−	−	−	−
Intellectual abilities	−	−	−	−	−	−	−	−	−	−	−	−	−	−	−	−
MRI of brain	CNH	CNH	NT	NT	CNH	NT	CNH	NT	NT	CNH	NT	CNH	NT	NT	NT	CNH
CT of temporal bone	−	−	NT	NT	−	NT	−	−	−	−	NT	−	NT	NT	NT	−

*Familial cases, representing the probands of the AN families. The other cases are sporadic cases.

†RefSeq: NM_004208.3, NP_ NP_004199.1, GRCh38/hg38 chrX: NC_000023.11 (130129362..130165887, complement).

‡The mutation of c.1030C>T (p.L344F) was detected not only in family 0223 but also in other three sporadic cases 1302, 1757 and 7187.

§The mutation of c.1264C>T (p.R422W) was detected not only in family 2724 but also in the two sporadic cases 3033 and 6962.

¶The degrees of hearing loss were evaluated based on the recommendations of the EU HEAR project, as described by Stephens (2001), and the detailed audiological data of the auditory neuropathy spectrum disorder cases are summarised in online supplementary table S9.

+/−, positive or negative finding; CNH, cochlear nerve hypoplasia; NT, not tested.

### Clinical manifestations of *AIFM1*-associated ANSD

The phenotype associated with the AUNX1-causing *AIFM1* p.R451Q mutation is characterised by childhood-onset ANSD and delayed peripheral sensory neuropathy presenting as extremity numbness, unsteadiness and areflexia.^25^ This clinical picture was seen with the other familial cases of *AIFM1* ANSD (0223, 7170, 2724 and 2423) and in some patients with sporadic *AIFM1* ANSD (table 1 and online supplementary table S9). The electrophysiological findings of 14 nerves in seven affected familial members were obtained (see online supplementary table S11). Nerve conduction studies demonstrated reduced sural, median and ulnar sensory NCVs or even absent responses. The reduced SCVs were always associated with reduced SNAPs (see online supplementary figure S3). The abnormal SEP results were also recorded, including no response or prolonged latency for the evoked potential P40 of tibial SEP, with or without prolonged latency of N9 potential of median SEP (see online supplementary figure S4). The data indicated that the patients might have demyelination changes in the peripheral sensory nerves. However, the MCVs and CMAPs of all patients were normal (see online supplementary figure S3). Needle EMG performed in these patients also showed normal values. There were no fibrillation, positive or fasciculation potentials and myotonic discharges were not observed. The patients also showed normal motor unit action potentials (see online supplementary figure S5). All of these findings suggested that the affected patients had evidence of peripheral sensory neuropathy but not motor neuropathy nor myopathy. The MMSE scores of patients III: 1, III: 9 and III: 11 from family 2423 were in the normal range (27–30): 28, 27 and 28, respectively with their corresponding educational backgrounds of junior college, high school and middle school. These normal scores (≥27) indicated normal cognitive function.

**Figure 4:**
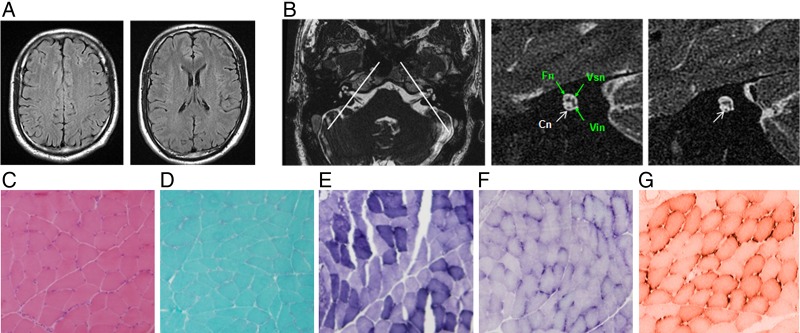
Brain MRI imaging and muscle biopsy immune-staining of the patient (III: 3) from family 0223. (A) Serial cerebral MRI with fluid-attenuated inversion recovery sequence demonstrates normal signal intensity in bilateral centrum semiovale (left panel) and periventricular and subcortical white matter (right panel). (B) Axial view of the cerebellopontine angle and the internal auditory canal (IAC) shows normal anatomy (left panel). The two white lines illustrate the plane prescribed for oblique plane sagittal images obtained perpendicular to the nerves of the IAC. The oblique plane sagittal image (3D-fast-spin echo sequence, middle panel) obtained on the left side demonstrates an abnormally small cochlea nerve (Cn, white arrow) but a normal size IAC with normal facial (Fn), superior (Vsn) and inferior (Vin) vestibular nerves (green arrows). The right Cn was symmetrically small (right panel, white arrow). (C–F) Immunohistochemical staining of muscle biopsy (left gastrocnemius) in patient III: 3 shows a few atrophic myofibers (H&E, C). No ragged red fibres (modified Gomori-trichrome, D), ragged blue fibres (succinate dehydrogenase, E) or targetoid fibres (nicotinamide adenine dinucleotide-tetrazolium reductase, F) are identified. There is no reduction or absence of cytochrome-*c*-oxidase histochemical reactions observed (G).

Re-examination of patients showed that both hearing impairment and sensory neuropathy slowly progress.^10^ ^25^ Although serial cerebral MRI in familial ANSD demonstrated normal signal intensity in the brain, inclined sagittal MRI of the internal auditory canals showed bilateral cochlea nerve hypoplasia (CNH), a finding consistent with the diagnosis of ANSD (figure 4A, B and table 1). No symptoms or signs of muscle wasting, weakness or atrophy were identified in *AIFM1* mutated patients, and muscle biopsy of the left gastrocnemius in an affected member of family 0223 (III: 2) revealed only a few atrophic myofibers (figure 4C–G). All patients with *AIFM1* mutations had normal serum levels of lactate dehydrogenase and creatine kinase.

### Impact on protein structure

We evaluated the functional effects of the 11 amino acid substitutions identified in this study using Polyphen2, SIFT, PROVEAN and Mutation Assessor.^26–30^ Nine variants were predicted to be likely pathogenic by at least three programs. (We considered the following predictions as pathogenic: Polyphen2, probably damaging; SIFT, damaging; PROVEA, deleterious; Mutation Assessor, medium/high functional impact). The exceptions, p.R422Q and p.I591M, were predicted pathogenic by two and one program, respectively (figure 3 and online supplementary table S12). Structural comparison of wild-type versus mutated AIFM1 protein showed that mutations in the two flavin adenine dinucleotide (FAD) and reduced nicotinamide adenine dinucleotide (NADH) domains have greater impact on the protein surface than mutations in C-terminus (see online supplementary figures S6 and S7).

### Histological findings

**Figure 5:**
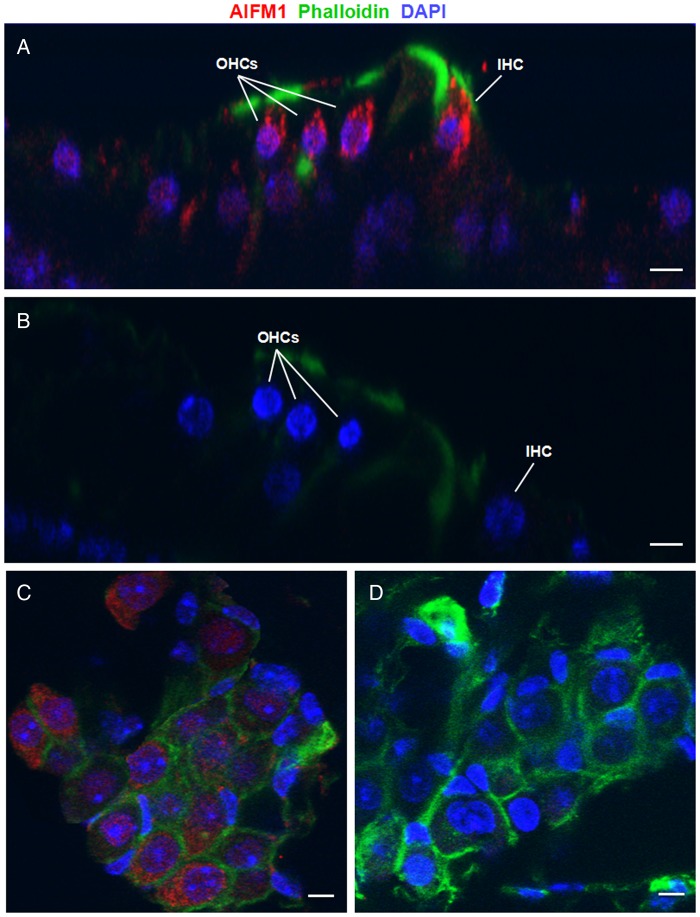
Localisation of AIFM1 in the murine inner ear by immunostaining with a monoclonal AIFM1 antibody. (A) Organ of Corti whole-mount preparation demonstrates AIFM1 (red) localisation to the cytoplasm of inner hair cell (IHC) and outer hair cells (OHCs), as well as the surrounding tissue. (B) Control organ of Corti tissue labelled with only secondary antibody and phalloidin (green). (C) Spiral ganglion whole-mount preparation shows AIFM1 (red) staining in spiral ganglion neurons. (D) Control spiral ganglion tissue labelled with secondary antibody and phalloidin (green). The scale bar indicates 15 µm in panels A and B, and 10 µm in panels C and D. DAPI, 4′,6-diamidino-2-phenylindole dihydrochloride.

Immunostaining of murine inner ear demonstrated ubiquitous localisation of AIFM1 in the inner, especially to the cytoplasm of IHC, OHCs and spiral ganglion neurons, consistent with a role in normal auditory function (figure 5 and online supplementary figures S8–S9).

## Discussion

In 2006, we mapped a novel X-linked auditory neuropathy locus (AUNX1) to chrXq23-q27.3 in a large five-generation Chinese family.^10^ Using WES and confirmatory segregation analysis, we now report a novel missense change, p.R451Q in *AIFM1*, as causally responsible for the phenotype in this family. Consistent with its playing a major role in ANSD, we have identified 10 other mutations in *AIFM1* in a cohort of familial and sporadic cases of ANSD of Chinese ethnicity. In all familial cases, the identified variants (p.T260A, p.L344F, p.R422W and p.R422Q) co-segregate with the auditory and peripheral sensory neuropathy. All 11 variants were absent in a screen of 500 normal-hearing, ethnicity-matched Chinese controls (750 X chromosomes) and 9 were classified as likely damaging by multiple bioinformatics programs (see online supplementary table S12). In aggregate, these data provide abundant compelling evidence to implicate *AIFM1* in X-linked recessive ANSD.

*AIFM1* encodes apoptosis-inducing factor 1, a flavoprotein located in the mitochondrial intermembrane space. AIFM1 has at least two functions.^31–33^ First, as a caspase-independent death effector, it mediates caspase-independent programmed cell death when translocating from mitochondria to the nucleus upon apoptotic stimuli. And second, as an FAD-dependent NADH oxidoreductase, it plays an important role in oxidative phosphorylation, redox control and respiratory chain activity in healthy cells. To date, *AIFM1* mutations have been associated with a severe mitochondrial encephalomyopathy (COXPD6, MIM# 300816; caused by p.R201del),^11^ prenatal ventriculomegaly (caused by p.G308E)^12^ and Cowchock syndrome (CMTX4, MIM# 310490; caused by p.E493V).^13^ Common features of these disorders are developmental disabilities such as mental retardation, motor dysfunction and muscle weakness, and abnormal MRI findings in brain.^11–13^ The AUNX1 phenotype is very different from these other phenotypes, as is the location of the causal mutations in the protein (see online supplementary table S13 and figure S10).

Based on phenotypic variability, it has been suggested that AIFM-related diseases have differing pathogenic mechanisms.^34^ In Cowchock syndrome, the p.E493V mutation alters the redox properties of the mutated protein, resulting in increased apoptosis.^13^ In COXPD6, in comparison, the R201del mutation reduces activity of respiratory chain complexes I–V and increases caspase-independent programmed cell death.^11^ Most of the 11 mutations identified in this study are located in the NADH and second FAD domains of AIFM1, which are essential for FAD-dependent NADH oxidoreductase.

Interestingly, in spite of the widespread expression of AIFM1 in murine inner ear, which is consistent with a role in normal auditory function, the mutated protein did not affect OHCs function as measured by distortion product otoacoustic emission responses (figure 5 and online supplementary table S9). In addition, while some patients with *AIFM1* mutations had MRI-documented CNH, the onset of hearing problems was typically during adolescence, suggesting that the hypoplasia represents late-onset and not congenital degeneration (see online supplementary figure S10; table 1).^35^ ^36^

ANSD is known to be an extremely complex disease that has congenital and acquired forms. Extensive clinical testing and genetic research are invaluable to elucidate underlying mechanisms and sites of pathology.^37–39^ Our finding of bilateral CNH in *AIFM1*-related ANSD implies that MRI screening may identify the site of lesion in some patients with this phenotype. Furthermore, it suggests that if CNH is an eventual common outcome cochlea implantation in patients with *AIFM1*-related ANSD may meet with limited success.^40–42^ In most patients, we were able to diagnose the other aspect of the phenotype, peripheral sensory neuropathy, by clinical and neurophysiological testing, although the symptoms of sensory neuropathy may occur many months or even years after the auditory neuropathy (see online supplementary table S11).

In conclusion, our study identifies *AIFM1* as a new causal gene associated with X-linked auditory neuropathy and delayed peripheral neuropathy. These results expand the spectrum of *AIFM1*-associated diseases to include ANSD. Because female carriers are unaffected, *AIFM1* should be considered in small pedigrees with apparent autosomal recessive ANSD if X-linked inheritance cannot be excluded. Further studies are required to determine the long-term benefit these patients may receive from cochlear implantation.

## Supplementary Material

Web figures

Web tables

## References

[1] Starr A, Picton TW, Sininger Y, Hood LJ, Berlin CI Auditory neuropathy. Brain 1996;119:741–53. 10.1093/brain/119.3.741 8673487

[2] Manchaiah VK, Zhao F, Danesh AA, Duprey R The genetic basis of auditory neuropathy spectrum disorder (ANSD). Int J Pediatr Otorhinolaryngol 2011;75:151–8. 10.1016/j.ijporl.2010.11.023 21176974

[3] Del Castillo FJ, Del Castillo I Genetics of isolated auditory neuropathies. Front Biosci (Landmark Ed) 2012;17:1251–65. 10.2741/3984 22201801

[4] Sanyelbhaa Talaat H, Kabel AH, Samy H, Elbadry M Prevalence of auditory neuropathy (AN) among infants and young children with severe to profound hearing loss. Int J Pediatr Otorhinolaryngol 2009;73:937–9. 10.1016/j.ijporl.2009.03.009 19409623

[5] El-Badry MM, McFadden SL Evaluation of inner hair cell and nerve fiber loss as sufficient pathologies underlying auditory neuropathy. Hear Res 2009;255:84–90. 10.1016/j.heares.2009.06.003 19531376 PMC2735340

[6] Giraudet F, Avan P Auditory neuropathies: understanding their pathogenesis to illuminate intervention strategies. Curr Opin Neurol 2012;25:50–6. 10.1097/WCO.0b013e32834f0351 22185903

[7] Ngo RY, Tan HK, Balakrishnan A, Lim SB, Lazaroo DT Auditory neuropathy/auditory dys-synchrony detected by universal newborn hearing screening. Int J Pediatr Otorhinolaryngol 2006;70:1299–306. 10.1016/j.ijporl.2005.12.004 16417926

[8] Beutner D, Foerst A, Lang-Roth R, von Wedel H, Walger M Risk factors for auditory neuropathy/auditory synaptopathy. ORL J Otorhinolaryngol Relat Spec 2007;69:239–44. 10.1159/000101545 17409783

[9] Declau F, Boudewyns A, Van den Ende J, van de Heyning P Auditory neuropathy: a challenge for diagnosis and treatment. B-ENT 2013;Suppl 21:65–79. 24383225

[10] Wang QJ, Li QZ, Rao SQ, Lee K, Huang XS, Yang WY, Zhai SQ, Guo WW, Guo YF, Yu N, Zhao YL, Yuan H, Guan J, Leal SM, Han DY, Shen Y AUNX1, a novel locus responsible for X linked recessive auditory and peripheral neuropathy, maps to Xq23-27.3. J Med Genet 2006;43:e33 10.1136/jmg.2005.037929 16816020 PMC2564562

[11] Ghezzi D, Sevrioukova I, Invernizzi F, Lamperti C, Mora M, D'Adamo P, Novara F, Zuffardi O, Uziel G, Zeviani M Severe X-linked mitochondrial encephalomyopathy associated with a mutation in apoptosis-inducing factor. Am J Hum Genet 2010;86:639–49. 10.1016/j.ajhg.2010.03.002 20362274 PMC2850437

[12] Berger I, Ben-Neriah Z, Dor-Wolman T, Shaag A, Saada A, Zenvirt S, Raas-Rothschild A, Nadjari M, Kaestner KH, Elpeleg O Early prenatal ventriculomegaly due to an AIFM1 mutation identified by linkage analysis and whole exome sequencing. Mol Genet Metab 2011;104:517–20. 10.1016/j.ymgme.2011.09.020 22019070

[13] Rinaldi C, Grunseich C, Sevrioukova IF, Schindler A, Horkayne-Szakaly I, Lamperti C, Landouré G, Kennerson ML, Burnett BG, Bönnemann C, Biesecker LG, Ghezzi D, Zeviani M, Fischbeck KH Cowchock syndrome is associated with a mutation in apoptosis-inducing factor. Am J Hum Genet 2012;91:1095–102. 10.1016/j.ajhg.2012.10.008 23217327 PMC3516602

[14] Li R, Li Y, Fang X, Yang H, Wang J, Kristiansen K, Wang J SNP detection for massively parallel whole-genome resequencing. Genome Res 2009;19:1124–32. 10.1101/gr.088013.108 19420381 PMC2694485

[15] McKenna A, Hanna M, Banks E, Sivachenko A, Cibulskis K, Kernytsky A, Garimella K, Altshuler D, Gabriel S, Daly M, DePristo MA The Genome Analysis Toolkit: a MapReduce framework for analyzing next-generation DNA sequencing data. Genome Res 2010;20:1297–303. 10.1101/gr.107524.110 20644199 PMC2928508

[16] Zhao Y, Zhao F, Zong L, Zhang P, Guan L, Zhang J, Wang D, Wang J, Chai W, Lan L, Li Q, Han B, Yang L, Jin X, Yang W, Hu X, Wang X, Li N, Li Y, Petit C, Wang J, Wang HY, Wang Q Exome sequencing and linkage analysis identified tenascin-C (TNC) as a novel causative gene in nonsyndromic hearing loss. PLoS One 2013;8:e69549 10.1371/journal.pone.0069549 23936043 PMC3728356

[17] Azaiez H, Booth KT, Bu F, Huygen P, Shibata SB, Shearer AE, Kolbe D, Meyer N, Black-Ziegelbein EA, Smith RJ TBC1D24 mutation causes autosomal-dominant nonsyndromic hearing loss. Hum Mutat 2014;35:819–23. 10.1002/humu.22557 24729539 PMC4267685

[18] Frommolt P, Abdallah AT, Altmüller J, Motameny S, Thiele H, Becker C, Stemshorn K, Fischer M, Freilinger T, Nürnberg P Assessing the enrichment performance in targeted resequencing experiments. Hum Mutat 2012;33:635–41. 10.1002/humu.22036 22290614

[19] Ding D, He J, Allman BL, Yu D, Jiang H, Seigel GM, Salvi RJ Cisplatin ototoxicity in rat cochlear organotypic cultures. Hear Res 2011;282:196–203. 10.1016/j.heares.2011.08.002 21854840 PMC3230738

[20] Ding D, Qi W, Yu D, Jiang H, Han C, Kim MJ, Katsuno K, Hsieh YH, Miyakawa T, Salvi R, Tanokura M, Someya S Addition of exogenous NAD+ prevents mefloquine-induced neuroaxonal and hair cell degeneration through reduction of caspase-3-mediated apoptosis in cochlear organotypic cultures. PLoS One 2013;8:e79817 10.1371/journal.pone.0079817 24223197 PMC3819247

[21] Korver AM, van Zanten GA, Meuwese-Jongejeugd A, van Straaten HL, Oudesluys-Murphy AM Auditory neuropathy in a low-risk population: a review of the literature. Int J Pediatr Otorhinolaryngol 2012;76:1708–11. 10.1016/j.ijporl.2012.08.009 22939591

[22] Shearer AE, Eppsteiner RW, Booth KT, Ephraim SS, Gurrola J, Simpson A, Black-Ziegelbein EA, Joshi S, Ravi H, Giuffre AC, Happe S, Hildebrand MS, Azaiez H, Bayazit YA, Erdal ME, Lopez-Escamez JA, Gazquez I, Tamayo ML, Gelvez NY, Leal GL, Jalas C, Ekstein J, Yang T, Usami S, Kahrizi K, Bazazzadegan N, Najmabadi H, Scheetz TE, Braun TA, Casavant TL, LeProust EM, Smith RJH Utilizing ethnic-specific differences in minor allele frequency to re-categorize reported pathogenic deafness variants. Am J Hum Genet 2014;95:445–53. 10.1016/j.ajhg.2014.09.001 25262649 PMC4185121

[23] Fukami M, Kirsch S, Schiller S, Richter A, Benes V, Franco B, Muroya K, Rao E, Merker S, Niesler B, Ballabio A, Ansorge W, Ogata T, Rappold GA A member of a gene family on Xp22.3, VCX-A, is deleted in patients with X-linked nonspecific mental retardation. Am J Hum Genet 2000;67:563–73. 10.1086/303047 10903929 PMC1287516

[24] Lahn BT, Page DC A human sex-chromosomal gene family expressed in male germ cells and encoding variably charged proteins. Hum Mol Genet 2000;9:311–19. 10.1093/hmg/9.2.311 10607842

[25] Wang Q, Gu R, Han D, Yang W Familial auditory neuropathy. Laryngoscope 2003;113:1623–9. 10.1097/00005537-200309000-00041 12972945

[26] Adzhubei IA, Schmidt S, Peshkin L, Ramensky VE, Gerasimova A, Bork P, Kondrashov AS, Sunyaev SR A method and server for predicting damaging missense mutations. Nat Methods 2010;7:248–9. 10.1038/nmeth0410-248 20354512 PMC2855889

[27] Kumar P, Henikoff S, Ng PC Predicting the effects of coding non-synonymous variants on protein function using the SIFT algorithm. Nat Protoc 2009;4:1073–81. 10.1038/nprot.2009.86 19561590

[28] Choi Y, Sims GE, Murphy S, Miller JR, Chan AP Predicting the Functional Effect of Amino Acid Substitutions and Indels. PLoS ONE 2012;7:e46688 10.1371/journal.pone.0046688 23056405 PMC3466303

[29] Reva B, Antipin Y, Sander C Determinants of protein function revealed by combinatorial entropy optimization. Genome Biol 2007;8:R232 10.1186/gb-2007-8-11-r232 17976239 PMC2258190

[30] Reva B, Antipin Y, Sander C Predicting the functional impact of protein mutations: application to cancer genomics. Nucleic Acids Res 2011;39:e118 10.1093/nar/gkr407 21727090 PMC3177186

[31] Hangen E, Blomgren K, Bénit P, Kroemer G, Modjtahedi N Life with or without AIF. Trends Biochem Sci 2010;35:278–87. 10.1016/j.tibs.2009.12.008 20138767

[32] Norberg E, Orrenius S, Zhivotovsky B Mitochondrial regulation of cell death: processing of apoptosis-inducing factor (AIF). Biochem Biophys Res Commun 2010;396:95–100. 10.1016/j.bbrc.2010.02.163 20494118

[33] Polster BM AIF, reactive oxygen species, and neurodegeneration: a “complex” problem. Neurochem Int 2013;62:695–702. 10.1016/j.neuint.2012.12.002 23246553 PMC3610861

[34] Delavallée L, Cabon L, Galán-Malo P, Lorenzo HK, Susin SA AIF-mediated caspase-independent necroptosis: a new chance for targeted therapeutics. IUBMB Life 2011;63:221–32. 10.1002/iub.432 21438113

[35] Roche JP, Huang BY, Castillo M, Bassim MK, Adunka OF, Buchman CA Imaging characteristics of children with auditory neuropathy spectrum disorder. Otol Neurotol 2010;31:780–8. 10.1097/MAO.0b013e3181d8d528 20593543 PMC3664382

[36] Young NM, Kim FM, Ryan ME, Tournis E, Yaras S Pediatric cochlear implantation of children with eighth nerve deficiency. Int J Pediatr Otorhinolaryngol 2012;76:1442–8. 10.1016/j.ijporl.2012.06.019 22921779

[37] Santarelli R Information from cochlear potentials and genetic mutations helps localize the lesion site in auditory neuropathy. Genome Med 2010;2:91 10.1186/gm212 21176122 PMC3025433

[38] Vlastarakos PV, Nikolopoulos TP, Tavoulari E, Papacharalambous G, Korres S Auditory neuropathy: endocochlear lesion or temporal processing impairment? Implications for diagnosis and management. Int J Pediatr Otorhinolaryngol 2008;72:1135–50. 10.1016/j.ijporl.2008.04.004 18502518

[39] Rapin I, Gravel J “Auditory neuropathy”: physiologic and pathologic evidence calls for more diagnostic specificity. Int J Pediatr Otorhinolaryngol 2003;67:707–28. 10.1016/S0165-5876(03)00103-4 12791445

[40] Kutz JW Jr, Lee KH, Isaacson B, Booth TN, Sweeney MH, Roland PS Cochlear implantation in children with cochlear nerve absence or deficiency. Otol Neurotol 2011;32:956–61. 10.1097/MAO.0b013e31821f473b 21659925

[41] Glastonbury CM, Davidson HC, Harnsberger HR, Butler J, Kertesz TR, Shelton C Imaging findings of cochlear nerve deficiency. AJNR Am J Neuroradiol 2002;23:635–43. 11950658 PMC7975095

[42] Valero J, Blaser S, Papsin BC, James AL, Gordon KA Electrophysiologic and behavioral outcomes of cochlear implantation in children with auditory nerve hypoplasia. Ear Hear 2012;33:3–18. 10.1097/AUD.0b013e3182263460 21750462

