# Supplementary material for: Mutations in apoptosis-inducing factor cause X-linked recessive auditory neuropathy spectrum disorder
Source: J Med Genet. 2015 May 18;52(8):523–31. doi: 10.1136/jmedgenet-2014-102961 (PMC4518735; doi:10.1136/jmedgenet-2014-102961)

Figure S1 The distribution of per-base sequencing depth in target regions for each sample in family 0223. Y-axis indicated the percentage of total target region under a given sequencing depth.

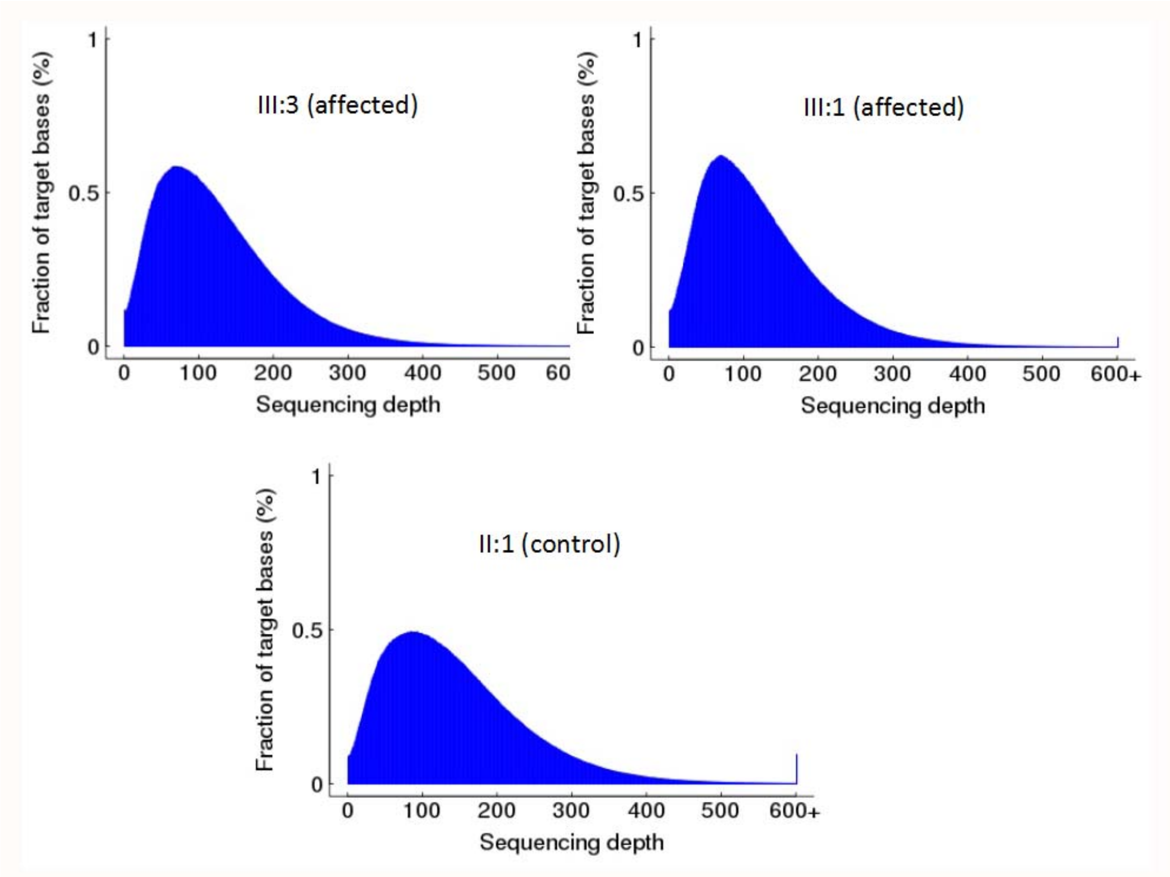

Figure S2 Cumulative depth distribution in target regions for each sample in family 0223. X-axis denotes sequencing depth, and Y-axis indicated the fraction of bases that achieves at or above a given sequencing depth.

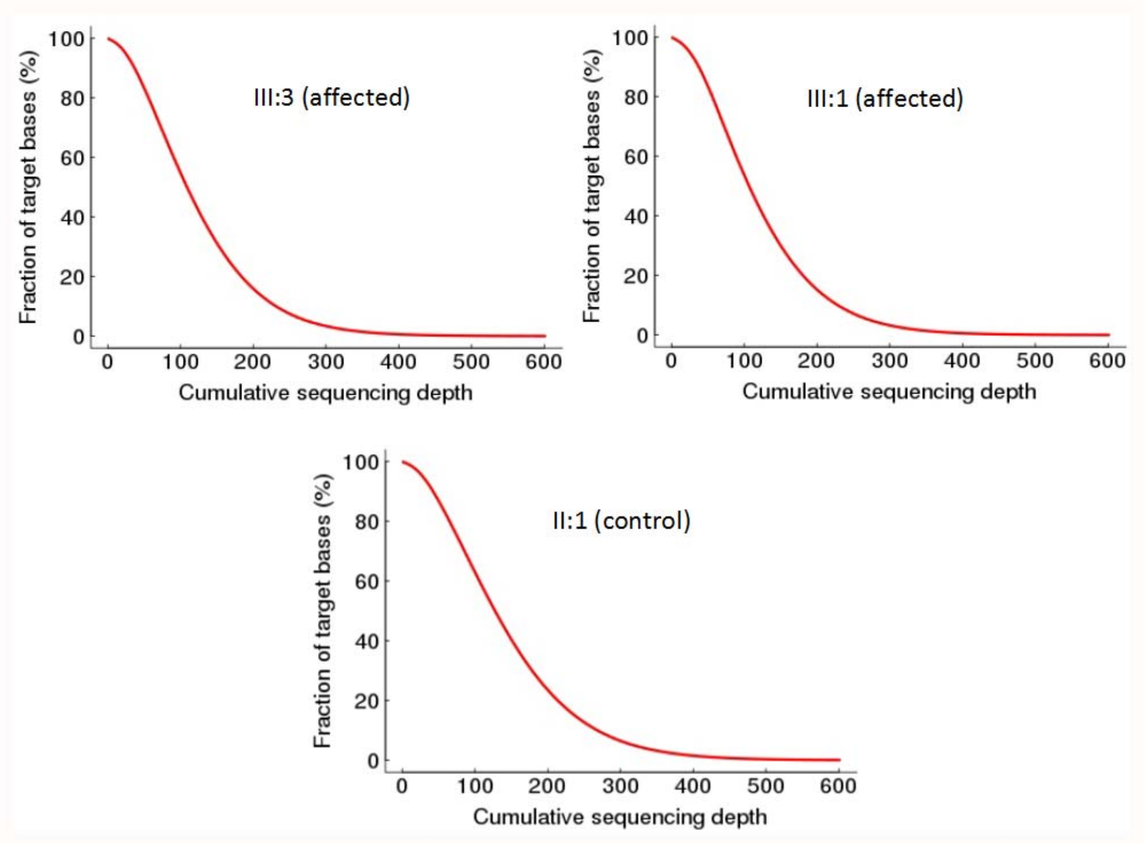

Figure S3 The CVs recordings in patient III: 9 of family 2423. The SCV of left suralis showed no response (upper panel). The MCV of left Peroneus was in normal range (lower panel).

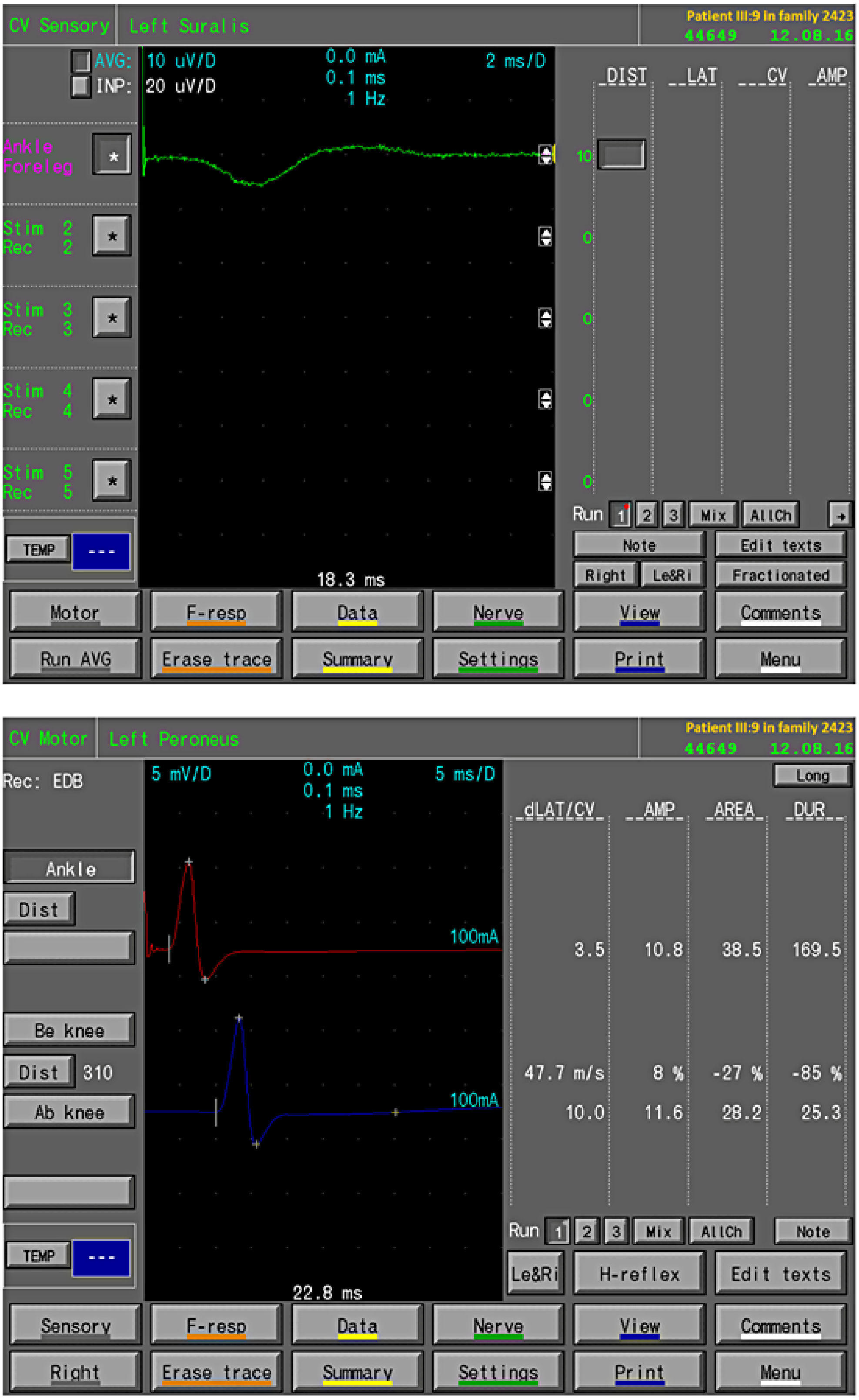

Figure S4 The SEP recordings in patient III: 9 of family 2423. Left tibial SEP at ankle showed no response for the evoked potential P40 (upper panel). Left median SEP at wrist showed prolonged latency of N9 potential (lower panel).

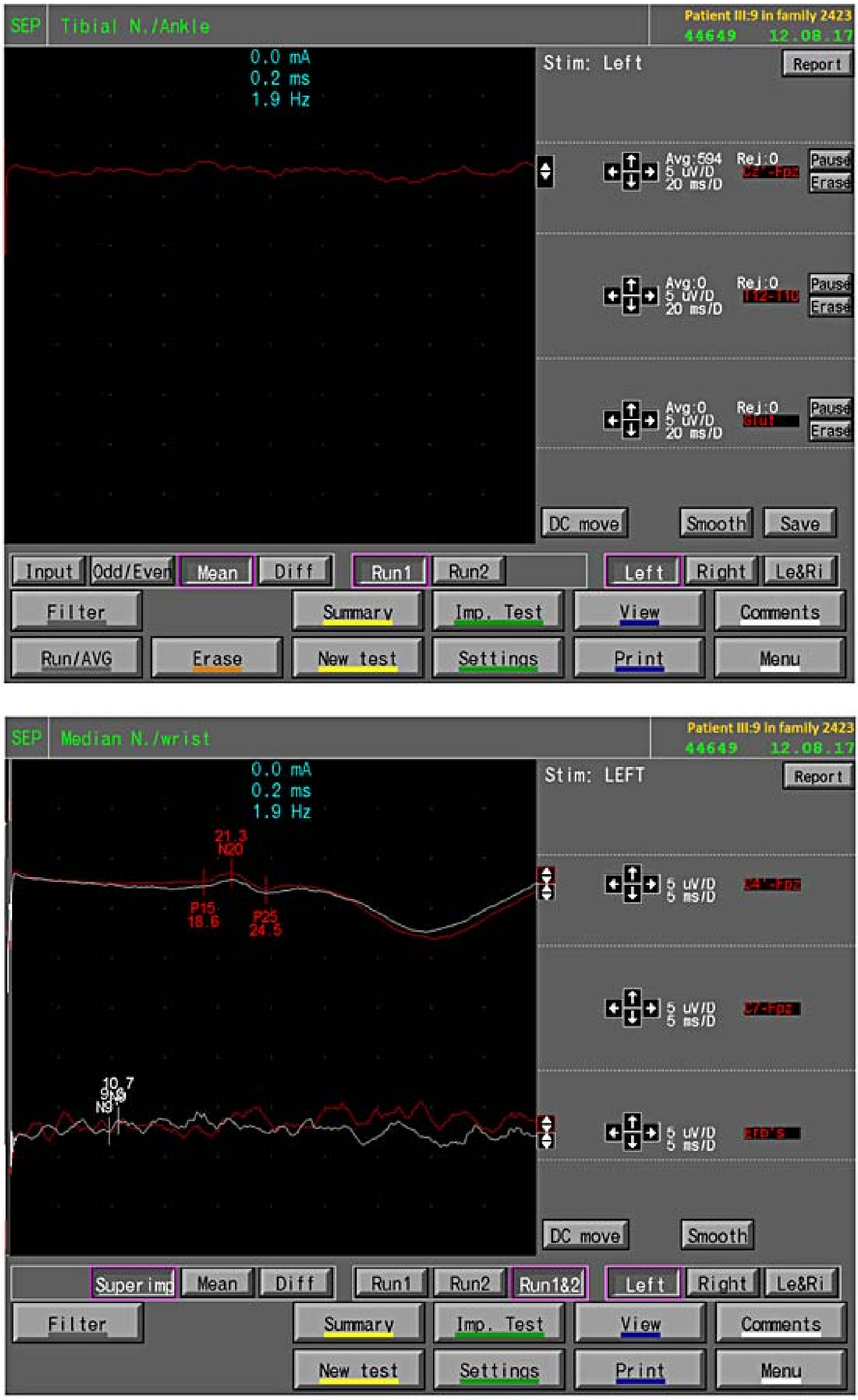

Figure S5 Normal MUPs recordings of patient III: 9 of family 2423.

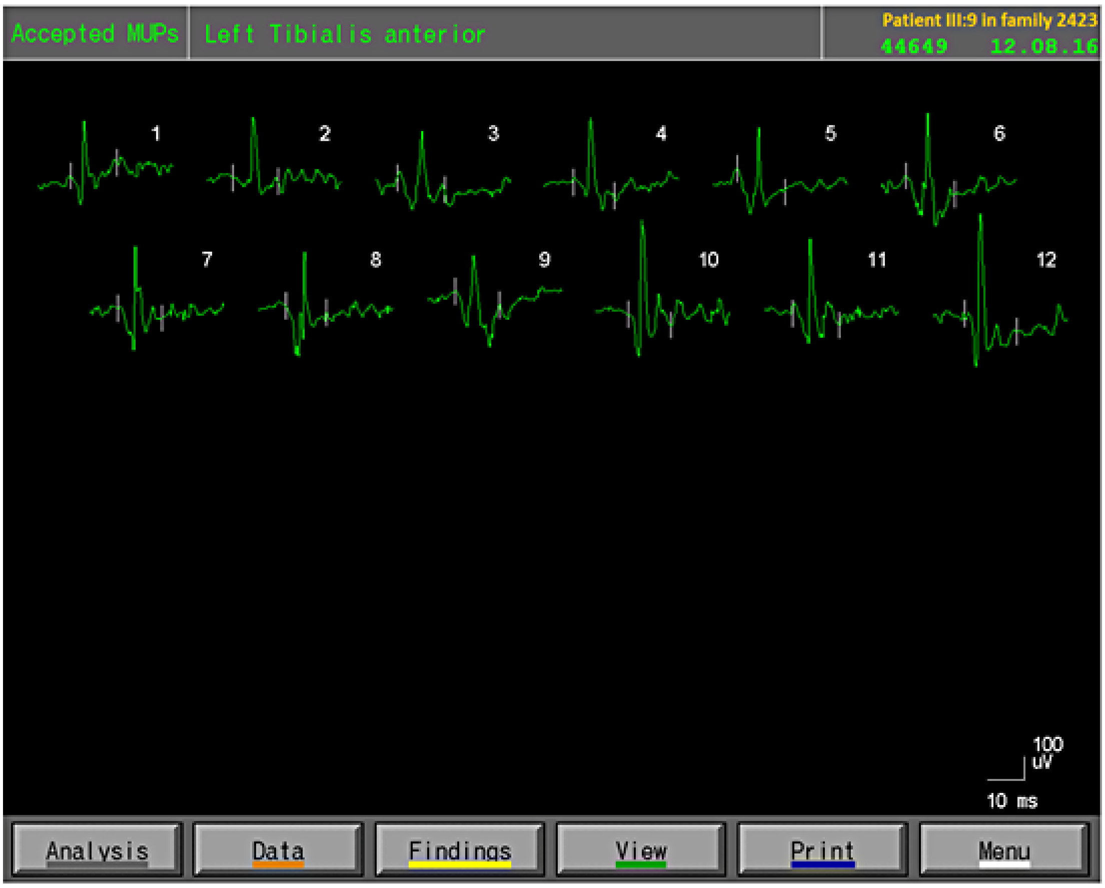

Figure S6 Structural comparison of wild-type and point mutants in the AIFM1 protein. (A and B) Location of the four mutant residues in the first FAD and the NADH domains of human AIFM1 (yellow arrows). Structure analysis of the wild-type AIFM1 (PDB ID: 4LII, generated by SWISS-MODEL program) indicates T260, L344 and G360 residues are located on the surface of mature protein and exposed to the solvent (white arrows). The mutated residue T260A in the first FAD domain and G360R in NADH domain (yellow circles) may have a greater impact on the protein functionality. (C and D) Location of the five identified amino acid substitutions (yellow arrows) in the second FAD domain. Their corresponding wild-type residues (white arrows) are all located on the surface of the protein. Among these mutated residues, R430C, R422W and P475L may have more functional effects (yellow circles). (reference: http://nar.oxfordjournals.org/content/early/2014/04/29/nar.gku340.abstract?keytype=ref&ijkey=FrXy4oQwsZzEAsw). All figures were generated using PyMOL (reference: http://www.pymol.org/citing).

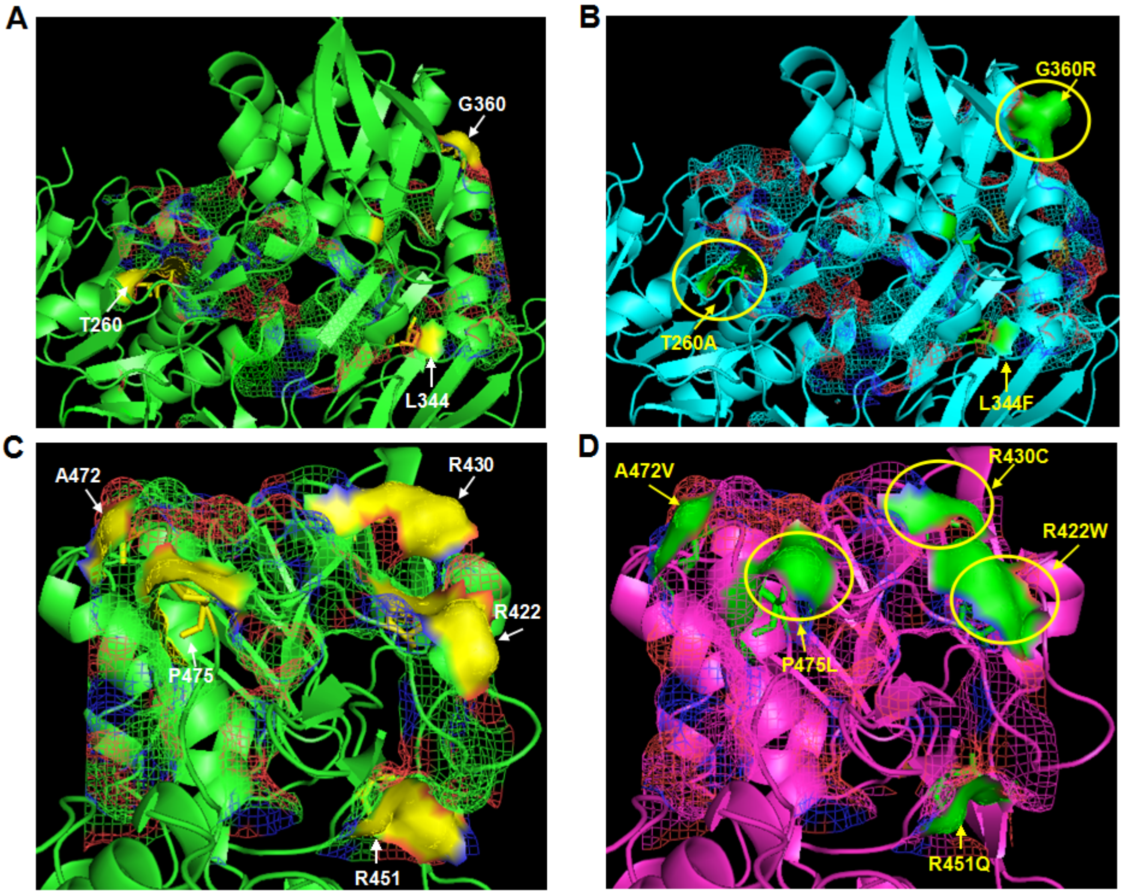

Figure S7 Locations of the two residues V498 and I591 (left panel) in C-terminus of AIFM1. Their mutated residues (right panel) have little effects on protein surface.

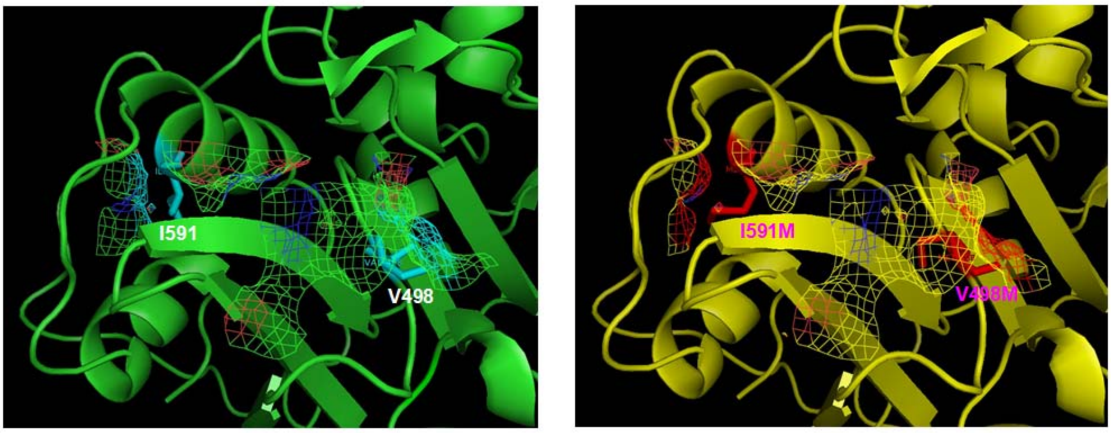

Figure S8 Localization of AIFM1 in stria vascularis. (A) Stria vascularis whole-mount preparation demonstrates the broad distribution of AIFM1. (B) The negative control without primary AIFM1 antibody. The scale bar indicates 15 μm.

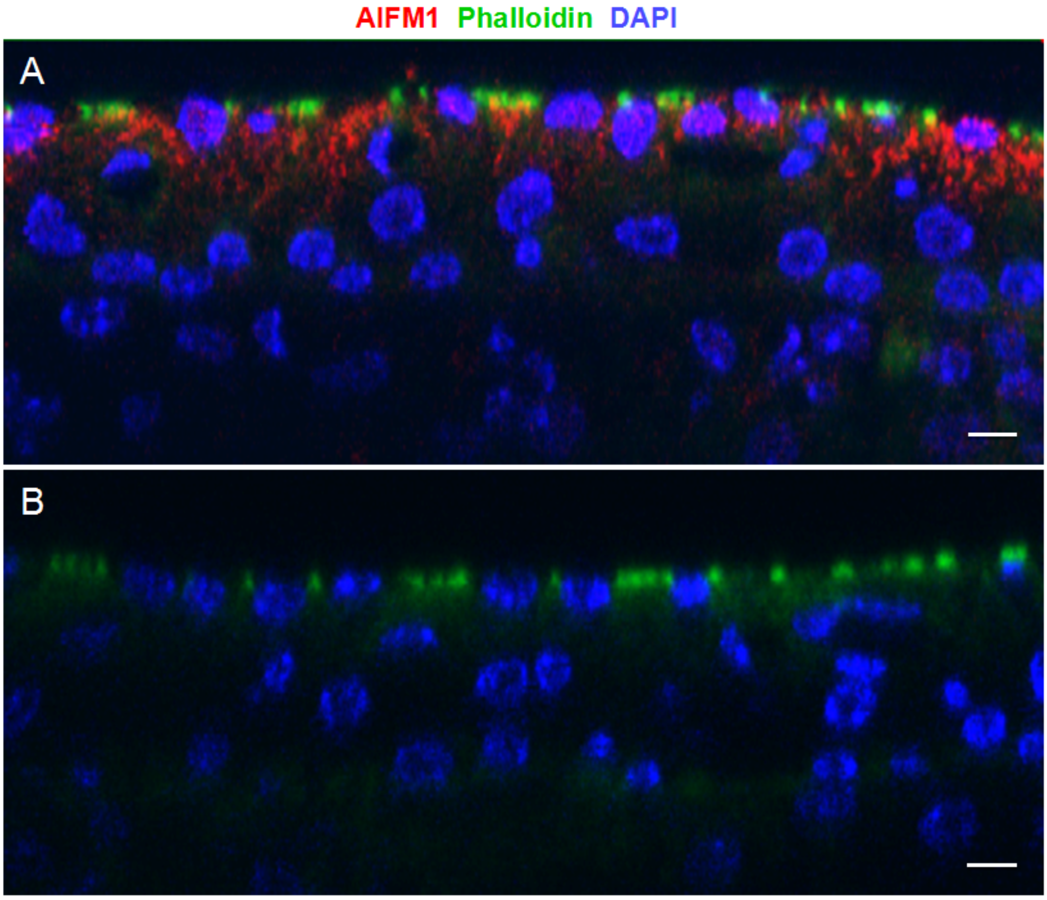

Figure S9 Localization of AIFM1 in vestibular tissue of murine inner ear. (A) Mouse macula of saccule labeled with AIFM1 antibody and phalloidin reveals AIFM1 localization to the hair cells. (B) The negative control without primary AIFM1 antibody. The scale bar indicates 15 μm.

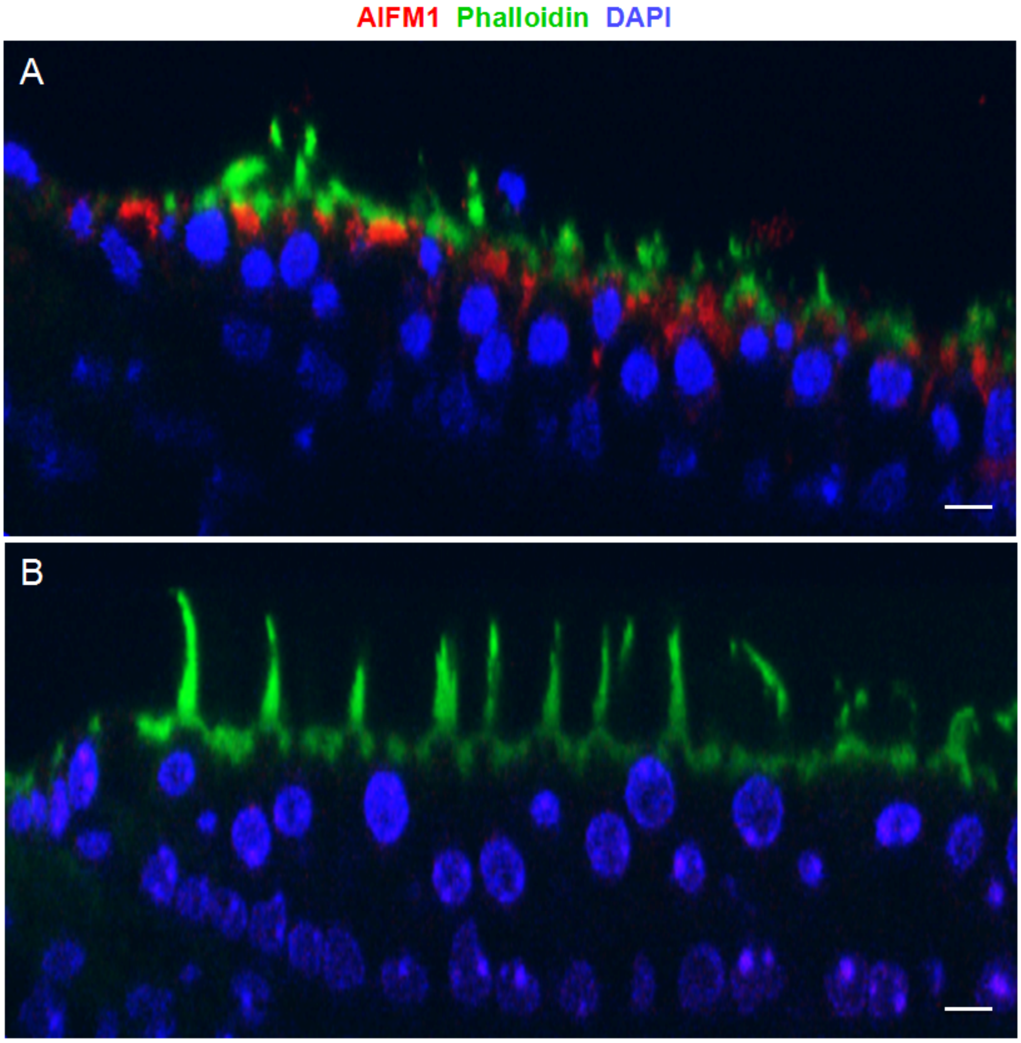

Figure S10 Age-of-onset distributions of auditory neuropathy (with or without peripheral neuropathy) patients with *AIFM1* mutations.

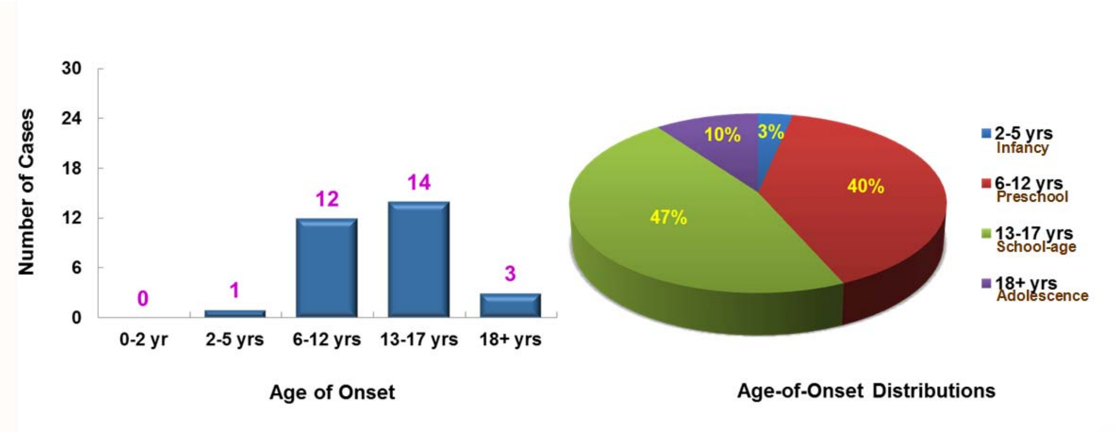

Supplement: Web figures [file jmedgenet-2014-102961-s1.pdf]
