# Supplementary material for: Mutations in apoptosis-inducing factor cause X-linked recessive auditory neuropathy spectrum disorder
Source: J Med Genet. 2015 May 18;52(8):523–31. doi: 10.1136/jmedgenet-2014-102961 (PMC4518735; doi:10.1136/jmedgenet-2014-102961)
Supplement: Web tables [file jmedgenet-2014-102961-s2.pdf]

Supplementary Tables (S1-S13):

Table S1 PCR primer pairs information for candidate genes screening.

Table S2 Summary of WES data for each sample of family 0223.

Table S3 Summary of SNPs and Indels for each sample of family 0223.

Table S4 Screening and identifying the causal genes by WES (SNPs$^{\#}$)

Table S5 Screening and identifying the causal genes by WES (Indels$^{\#}$)

Table S6 Screening and identifying the causal genes by WES (Functional SNPs$^{\#}$)

Table S7 Screening and identifying the causal genes by WES (Functional Indels$^{\#}$)

Table S8 Candidate variants shared by two affected individuals of family 0223

Table S9 Audiological test data of cases with AIFM1 mutations

Table S10 The allele frequency of total eleven identified AIFM1 variants from genetic variation databases.

Table S11 Nerve conduction velocities in seven familial ANSD cases accompanied by late-onset peripheral neuropathy.

Table S12 Missense mutations pathogenicity prediction of AIFM1 using in silico bioinformatic tools.

Table S13 Different phenotypes of diseases caused by AIFM1 mutations.

Table S1 PCR primer pairs information for candidate genes screening

Candidate genes	Screened region	Forward Primer (5'-3')	Reverse Primer (5'-3')	PCR Products Size (bp)
AIFM1	Exon1	GCTACGCTGTTGTGAGATGCT	GCCGACTACTGGGTTCAAATC	788
AIFM1	Exon2	ATCTGTGGGCAATAAGTCT	AACATAGTGGCTTTCAAGT	506
AIFM1	Exon3	GAGCCTAAAAATCTGAAACT	ATAACTTTCCCTTTGTGAG	393
AIFM1	Exon4 & 5	GTGGCAAAGAATCATCTGAG	CTTGCCCTTTGTAGACTGTT	616
AIFM1	Exon6	CCCCAAGTTGAGAACCACT	GGAAACACACATCACCATACT	358
AIFM1	Exon7	TTGGGGTGGTGATGGAAAT	GAAGGCTGGACTCTAAAACT	337
AIFM1	Exon8	ACCCCTTGAAGACAGACTC	TGGGGACTGCAAGATTATAC	362
AIFM1	Exon9	TGCCCTGACAACCAAAAAT	ATCCTGCCAAACACATCTCT	465
AIFM1	Exon10	CCTGCTGCTCCTTTACTTCT	ACTGGAGAATGGTGGAAACA	325
AIFM1	Exon11	TTCCACCATTCTCCAGTCAG	GCAAGGGGAGTGGAGAAC	374
AIFM1	Exon12	GTGGTGGAGGCTTATGAAAT	CTCAGCCTCCAAACACTCT	431
AIFM1	Exon13	TGAGCCCCCAAAGTTTAT	ATCTCCATTCATTCACCTAGT	507
AIFM1	Exon14	TGTGCTACCGTGTCATTCCT	TGCCAAATCTCAGACCACT	361
AIFM1	Exon15	GGAGGGAAGTTTAGGGTCAG	GGCACCCGATGAAGTTACAG	521
AIFM1	Exon16	CGGCTTAGAAACATTCCTG	AGGAGTTTTGCGTCTGGAGT	707
HS6ST2	Exon6	CAAAAGCGTATTGAGGGACTG	CAGGTTCTGATTGGCATTCG	246

Table S2 Summary of WES data for each sample of family 0223

Exome Capture Statistics	II: 1 (control)	III: 1 (affected)	III: 3 (affected)
Initial bases on target	50390601	50390601	50390601
Initial bases on or near target	124292823	124292823	124292823
Total effective reads	137440891	115293941	119954353
Total effective yield(Mb)	12145.66	10197.76	10602.99
Number of reads uniquely mapped to genome	131369753	110497810	114693978
Number of reads uniquely mapped to target	94420833	80077072	81339103
Effective sequences on target(Mb)	7258.88	6150.15	6240.29
**Average sequencing depth on target**	**144.05**	**122.05**	**123.84**
**Coverage of target region**	**99.90%**	**99.80%**	**99.80%**
Average read length(bp)	88.37	88.45	88.39
Mismatch rate in target region	0.30%	0.29%	0.30%
Fraction of effective bases on target	59.80%	60.30%	58.90%
Fraction of effective bases on or near target	80.10%	80.60%	79.50%
Fraction of uniquely mapped on target	71.90%	72.50%	70.90%
Fraction of target covered >=20x	97.10%	96.40%	96.40%
Fraction of target covered >=10x	98.90%	98.60%	98.60%
Fraction of target covered >=4x	99.60%	99.50%	99.50%
Coverage of flanking region	98.40%	98.00%	98.30%

Fraction of flanking region covered >=20x	50.50%	44.80%	47.50%
Fraction of flanking region covered >=10x	71.30%	66.20%	69.30%
Fraction of flanking region covered >=4x	90.10%	87.50%	89.30%
Mapping rate	99.41%	99.50%	99.41%
Duplicate rate	8.85%	8.20%	8.42%
Gender test result	Male	Male	Male

Table S3 Summary of SNPs and Indels for each sample of family 0223

Items/Samples	0400223-1	0400223-2	400223
**Total SNPs**	**90642**	**86156**	**89213**
Novel	876	786	832
Hom	41693	40068	41008
Het	48949	46088	48205
Synonymous	10122	9973	10080
Missense	9006	8818	8985
Stopgain	56	63	63
Stoploss	28	32	30
Startgain	270	236	268
Startloss	19	18	21
Exonic	20832	20485	20790
Splicing	79	68	74
**Total Indels**	**12091**	**11278**	**11481**
Novel	732	587	617
Hom	5666	5428	5533
Het	6425	5850	5948
Frameshift	226	208	209
Non-frameshift Insertion	82	86	85

Non-frameshift Deletion	92	89	83
Non-frameshift codon substitution	0	0	0
Non-frameshift codon substitution plus Insertion	21	17	20
Non-frameshift codon substitution plus Deletion	53	63	50
Stopgain	2	2	2
Stoploss	7	8	8
Startgain	0	0	0
Startloss	4	5	5
Exonic	751	735	731
Splicing	55	55	56

Note: The value of the first column takes the following precedence: exonic = splicing >ncRNA>> UTR5/UTR3 > intron > upstream/downstream >intergenic.

(1) Hom: homozygous; Het: heterozygous.

(2) Exonic here refers only to coding exonic portion, but not UTR portion.

(3) For SNPs, stopgain means that a nonsynonymous SNV that lead to the immediate creation of stop codon at the variant site. Meanwhile stoploss means that lead to the immediate elimination of stop codon at the variant site.

(4) Splicing is defined as variant that is within 2-bp away from an exon/intron boundary.

(5) Frameshift mutation means that an insertion/deletion of one or more nucleotides that cause frameshift changes in protein coding sequence.

(6) Nonframeshift mutation means that an insertion/deletion of 3 or multiples of 3 nucleotides that do not cause frameshift changes in protein coding sequence.

(7) Non-frameshift codon substitution means that one or many codons are changed, an MNP of size multiple of 3; Non-frameshift codon substitution plus Insertion (Deletion) means that One codon is changed and one or many codons are inserted (deleted). An insert (deletion) of size multiple of three, not at codon boundary.

(8) For Indels, stopgain means that a frameshift insertion/deletion, nonframeshift insertion/deletion or block substitution that lead to the immediate creation of stop codon at the variant site. For frameshift mutations, the creation of stop codon downstream of the variant will not be counted as "stopgain".Meanwhile stoploss means that lead to the immediate elimination of stop codon at the variant site.

Table S4 Screening and identifying the causal genes by WES (SNPs[#])

Feature SNP	II: 1 (control)	III: 1 (affected)	III: 3 (affected)
Total SNPs	90642	86156	89213
Filtered_dbSNP	74850	71038	73573
Filtered_dbSNP_1000G	3346	3138	3237
Filtered_dbSNP_1000G_Hapmap	3113	2921	3012
Filtered_dbSNP_1000G_Hapmap_YH	2744	2549	2640
Filtered_dbSNP_1000G_Hapmap_YH_Ctr1	0	1220	1318
Share all cases		561	

[#]Function: missense|readthrough|nonsense|spliceSite|synonymous-coding|5-UTR|3-UTR|intron|intergenic

Table S5 Screening and identifying the causal genes by WES (Indels#)

Feature indels	II: 1 (control)	III: 1 (affected)	III: 3 (affected)
Total Indels	3379	3266	3273
Filtered_dbIndel	2275	2193	2217
Filtered_dbIndel_1000G	2043	1961	1985
Filtered_dbIndel_1000G_Hapmap	2043	1961	1985
Filtered_dbIndel_1000G_Hapmap_YH	2042	1960	1984
Filtered_dbIndel_1000G_Hapmap_YH_Ctrl	0	462	455
Share all cases		245	

#Function: frameshift|cds-indel|spliceSite|5-UTR|3-UTR|intron|promoter|intergenic

Table S6 Screening and identifying the causal genes by WES (Functional SNPs[#])

Feature SNP	II: 1 (control)	III: 1 (affected)	III: 3 (affected)
Functional_SNPs	12540	12344	12541
Filtered_dbSNP	10349	10172	10371
Filtered_dbSNP_1000G	599	609	614
Filtered_dbSNP_1000G_Hapmap	575	586	590
Filtered_dbSNP_1000G_Hapmap_YH	525	532	537
Filtered_dbSNP_1000G_Hapmap_YH_Ctr1	0	260	285
Share all cases		129	

[#]Function: missense|readthrough|nonsense|spliceSite

Table S7 Screening and identifying the causal genes by WES (Functional Indels#)

Feature indels	II: 1 (control)	III: 1 (affected)	III: 3 (affected)
Functional Indels	1170	1141	1148
Filtered_dbIndel	769	754	769
Filtered_dbIndel_1000G	706	688	696
Filtered_dbIndel_1000G_Hapmap	706	688	696
Filtered_dbIndel_1000G_Hapmap_YH	705	687	695
Filtered_dbIndel_1000G_Hapmap_YH_Ctrl	0	169	158
Share all cases		84	

#Function: frameshift|cds-indel|spliceSite

Table S8 Candidate variants shared by two affected individuals of family 0223

Chromosome	Position	Reference	Change	Gene	Codon	Substitution
ChrX	129271098	C	T	AIFM1	Ctc1030Ttc	L344F
Chr4	120550141	-3GCA	/	PDE5A	5-UTR	Deletion
Chr3	73651621	-1A	/	PDZRN3	Splice site	Deletion
Chr12	42853058	-1T	/	PRICKLE1	3-UTR	Deletion
Chr2	202344179	-1T	/	STRADB	NR_exon	Deletion

/, not presenting the base substitution; NR_exon, non-coding exon variant.

Table S9 Audiological test data of cases with *AIFM1* mutations

Case ID	Age of onset (yrs)	Age at test (yrs)	Hearing level		SDS (%)		Tymp		Stapedial reflex		ABR[c]		DPOAE[d] (kHz)		ECochG[e]	
			PTA[b]	Audiogram	L	R	L	R	L	R	L	R	L	R	L	R
7170[a]	19	20	28.75	up-slope	48	84	A	As	/	/	NR	NR	0.75-4, 8	0.75-4, 8	1.02	0.64
0223[a]	16	27	35.00	up-slope	12	20	A	A	/	/	NR	NR	0.5-8	0.5-8	NR of AP	1.47
1302	17	20	36.25	up-slope	60	52	A	A	/	/	NR	NR	0.75-8	0.75-8	NA	NA
1757	19	19	32.5	up-slope	84	92	A	A	/	/	Vt=6.35ms,miss- -ing waves I-III		0.5-8	0.75-8	NA	NA
7187	13	25	41.25	up-slope	NA	NA	A	A	/	/	NR	NR	0.75-8	0.75-6	0.56	0.77
1747	14	17	46.25	up-slope	0	20	A	A	/	/	NR	NR	0.5-8	0.5-3, 6	0.96	1.72
2724[a]	18	31	48.75	up-slope	36	12	A	A	/	/	NR	NR	0.5-8	0.5-8	1.12	1.76
3033	13	16	38.75	inverted U-shape	64	52	A	A	/	/	NR	NR	0.75-8	1-3, 8	0.83	NR of AP
6962	15	20	36.25	up-slope	60	52	A	A	/	/	NR	NR	0.75-8	0.75-8	NA	NA
2423[a]	14	36	47.50	up-slope	16	20	A	A	/	/	NR	NR	0.5-8	0.5-3, 6, 8	0.83	1.00
0077	7	15	32.50	U-shape	NA	NA	A	A	/	/	NR	NR	0.5-8	0.5-8	NA	NA
AUNX1[a]	16	27	43.75	flat	0	0	A	A	/	/	NR	NR	0.5-3, 8	0.5-8	>1	0.71
1806	8	20	40.00	up-slope	16	44	A	A	/	/	NR	NR	0.5, 1-8	0.5-8	0.55	0.68
0046	14	24	56.25	up-slope	NA	NA	A	A	/	/	NR	NR	0.5, 1.5-8	0.5-8	>1	>1
4678	5	14	51.25	up-slope	NA	NA	A	A	/	/	NR	NR	0.75-8	0.75-8	NA	NA

3305	11	15	32.50	U-shape	48	56	A	A	/	/	NR	NR	0.75-8	0.75-8	1.22	2.60

[a] the familial case, representing the proband of the AN family.

[b] PTA, pure-tone air-conduction averages (0.5, 1, 2 and 4 kHz) for the better-hearing ear of the affected subjects (dB HL).

[c]. ABR was evoked by the click stimulus at maximum intensity level (100 dB nHL).

[d] the frequencies with DPOAE response in normal level.

[e] Click-evoked ECochG was recorded at the stimulating intensity of 100 dB nHL, and analyzed by the amplitude ratio of –SP/AP.

AN, auditory neuropathy; SDS, the speech discrimination score; Tymp, tympanometry; /, absent of stapedial reflex; ABR, auditory brainstem response; nHL, normal hearing level; DPOAE, distortion product otoacoustic emission; ECochG, electrocochleogram; SP/AP, summating potential/action potential; NA, not available; NR, no response; Vt, wave V potential time.

Table S10 The allele frequency of total eleven identified *AIFM1* variants from genetic variation databases

Variants	1000G_ALL	1000G_EAS	1000G_AFR	1000G_AMR	1000G_EUR	ESP 6500_ALL	ESP 6500_AA	ESP 6500_EA	dbSNP (142)	Controls*
c.778A>G (p.T260A)	—	—	—	—	—	—	—	—	—	—
c.1030C>T (p.L344F)	0.000529801	0.002	—	—	—	—	—	—	NA (rs184474885)	—
c.1078G>C (p.G360R)	—	—	—	—	—	—	—	—	—	—
c.1264C>T (p.R422W)	—	—	—	—	—	—	—	—	—	—
c.1265G>A (p.R422Q)	—	—	—	—	—	—	—	—	—	—
c.1288C>T (p.R430C)	—	—	—	—	—	—	—	—	—	—
c.1352G>A (p.R451Q)	—	—	—	—	—	—	—	—	—	—
c.1415C>T (p.A472V)	—	—	—	—	—	—	—	—	—	—
c.1424C>T (p.P475L)	—	—	—	—	—	—	—	—	—	—
c.1492G>A (p.V498M)	—	—	—	—	—	—	—	—	—	—
c.1773C>G (p.I591M)	—	—	—	—	—	—	—	—	—	—

* Controls in this study included 500 healthy ethnicity-matched individulas (125 males and 125 females with normal hearing, 750 X chromosomes).

1000G_ALL, allele frequency in all populations in the 1000 Genomes project; 1000G_EAS, allele frequency in East Asian populations in the 1000 Genomes project; 1000G_AFR, allele frequency in African populations in the 1000 Genomes project; 1000G_AMR, allele frequency in American populations in the 1000 Genomes project; 1000G_EUR, allele frequency in European populations in the 1000 Genomes project; ESP 6500_ALL, allele frequency in all subjects in the NHLBI ESP 6500 exomes; ESP 6500_AA, allele frequency in African Americans in the NHLBI ESP 6500 exomes; ESP 6500_EA, allele frequency in European Americans in the NHLBI ESP 6500 exomes; dbSNP (142), the database of Short Genetic Variations (human Build 142); —, negative finding; NA, the allele frequency is not available.

Table S11 Nerve conduction velocities in seven familial ANSD cases accompanied by late-onset peripheral neuropathy

Family	Patient	Course of AN/PN (yrs)	Side (L/R)	Tibial motor		Peroneal motor		Median motor		Ulnar motor		Sural sensory		Median sensory		Ulnar sensory	
				CV	Amp	CV	Amp	CV	Amp	CV	Amp	CV	Amp	CV	Amp	CV	Amp
AUNX1	III:22	11/1	L	44.2	*8.5*	45.8	*4.4*	56.4	*17.2*	57.8	*7.6*	NR	NR	47.1	3.7	44.8	3.1
					**9.3**		**5.8**		**17.5**		**7.5**						
			R	44.7	9.9	45.8	*4.4*	57.9	*7.5*	55.3	*7.0*	NR	NR	NR	NR	38.2	0.7
					**10.7**		**4.3**		**8.1**		**7.4**						
	III:23	19/0.5	L	43.5	*6.9*	43.8	*3.6*	58.3	*13.1*	56.5	*7.6*	NR	NR	NR	NR	NR	NR
					**7.6**		**3.9**		**13.6**		**7.8**						
			R	44.6	*4.7*	43.1	*4.3*	NT	*NT*	NT	*NT*	NR	NR	NR	NR	NR	NR
					**4.6**		**4.5**		**NT**		**NT**						
7170	IV:2	19/2	L	48.8	*21.4*	48.5	*4.5*	59.0	*18.2*	55.6	*18.3*	NR	NR	NR	NR	NR	NR
					**25.0**		**3.2**		**18.7**		**18.6**						
			R	52.5	*20.1*	50.8	*5.5*	NT	*NT*	NT	*NT*	40.5	2.3	42.1	0.8	NR	NR
					**23.5**		**6.4**		**NT**		**NT**						
2724	III:4	19/10	L	51.3	*10.1*	53.6	*6.6*	61.1	*21.0*	57.8	*12.2*	NR	NR	57.7	0.9	NR	NR
					**10.1**		**6.9**		**21.4**		**12.6**						
			R	52.0	*8.0*	50.0	*8.3*	NT	*NT*	NT	*NT*	NR	NR	60.0	1.0	NR	NR
					**11.7**		**6.0**		**NT**		**NT**						
2423	III:1	29/27	L	48.2	*15.1*	45.2	*12.4*	53.7	*13.6*	54.9	*15.0*	37.5	3.3	47.1	3.3	32.6	2.6
					**17.4**		**13.5**		**14.7**		**17.4**						
			R	47.1	*18.8*	45.2	*9.5*	55.0	*16.1*	56.0	*15.8*	42.9	2.0	51.6	3.0	41.2	2.4

				**20.5**		**10.0**		**16.4**		**16.0**						
III:9	22/4	L	43.3	*9.2*	46.3	*18.2*	56.4	*20.7*	60.0	*12.8*	NR	NR	32.7	1.2	32.4	0.9
				**10.6**		**18.1**		**22.2**		**12.9**						
		R	44.3	*7.5*	46.3	*18.2*	56.4	*19.3*	55.1	*14.7*	NR	NR	NR	NR	34.3	1.5
				**9.9**		**14.2**		**21.3**		**15.5**						
	28/10	L	47.0	*7.8*	47.7	*11.6*	66.7	*20.6*	56.2	*10.7*	NR	NR	44.4	1.5	37.5	0.4
				**10.3**		**10.8**		**21.2**		**10.8**						
		R	44.8	*8.2*	49.2	*11.8*	NT	*NT*	NT	*NT*	NR	NR	NT	NT	NT	NT
				**9.5**		**9.3**		**NT**		**NT**						
III:11	18/5	L	48.7	*16.2*	48.4	*28.7*	56.4	*18.3*	55.1	*17.1*	NR	NR	51.6	3.5	52.0	2.1
				**18.7**		**30.4**		**19.2**		**17.4**						
		R	45.8	*20.7*	48.4	*19.6*	55.0	*16.7*	52.9	*21.1*	40.5	1.1	50.0	2.1	46.4	3.6
				**22.0**		**24.1**		**16.7**		**23.2**						

The italic represents proximal stimulation, and the boldface represents distal stimulation. PN, late-onset peripheral neuropathy; L/R, left/right; CV, conductin velocity (m/s); Amp, amplitude (mV for motor; μV for sensory); NR, no response; NT, not tested. The abnormal values are shown in red. Normal CVs: motor tibial ≥ 40.0, peroneal ≥ 40.0, median ≥ 50.0, and ulnar ≥ 50.0; sensory sural ≥ 50.0, median ≥ 50.0, and ulnar ≥ 50.0. Normal amplitudes: motor tibial ≥ 5.0, peroneal ≥ 3.0, median ≥ 5.0, and ulnar ≥ 5.0; sensory sural ≥ 2.0, median ≥ 2.0, and ulnar ≥ 2.0.

Table S12 Missense mutations pathogenicity prediction of *AIFM1* using in silico bioinformatic tools

Mutations	Associated case ID	Polyphen2 prediction				SIFT prediction		PROVEAN prediction		Mutation assessor		Related protein domain
		Result	Score	Sensitivity	Specitivity	Result	Score	Result[a]	Score	Func. impact[b]	Score	
p.T260A	Family 7170	Possibly damaging	0.858	0.830	0.930	Damaging	0.010	Deleterious	-4.631	High	4.010	FAD-binding
p.L344F	1302, 1757, 7187 and family 0223	Possibly damaging	0.846	0.83	0.930	Tolerated	0.150	Deleterious	-3.554	Medium	2.150	NADH-binding
p.G360R	1747	Probably damaging	1.000	0.000	1.000	Damaging	0.000	Deleterious	-7.948	High	3.955	NADH-binding
p.R422W	3033, 6962 and family 2724	Probably damaging	1.000	0.000	1.000	Tolerated	0.090	Deleterious	-4.281	Medium	2.740	FAD-binding
p.R422Q	Family 2423	Probably damaging	1.000	0.000	1.000	Tolerated	0.640	Neutral	-1.974	Medium	2.045	FAD-binding
p.R430C	0077	Probably damaging	1.000	0.000	1.000	Damaging	0.010	Deleterious	-7.948	Medium	2.390	FAD-binding
p.R451Q	Family AUNX1	Probably damaging	1.000	0.000	1.000	Damaging	0.000	Deleterious	-3.970	High	3.520	FAD-binding
p.A472V	1806	Benign	0.170	0.920	0.870	Damaging	0.030	Deleterious	-3.373	Medium	2.750	FAD-binding
p.P475L	0046	Possibly damaging	0.517	0.880	0.900	Damaging	0.020	Deleterious	-8.993	Medium	2.690	FAD-binding
p.V498M	4768	Probably damaging	1.000	0.000	1.000	Damaging	0.020	Deleterious	-2.664	Medium	2.300	C-terminal
p.I591M	3305	Possibly damaging	0.898	0.820	0.940	Tolerated	0.110	Neutral	-2.093	Low	1.910	C-terminal

[a] For the PROVEAN prediction result, the cutoff score was -2.500.

[b] Func. impact, functional impact of a variant: predicted functional (high, medium) and predicted non-functional (low, neutral).

Table S13 Different phenotypes of diseases caused by *AIFM1* mutations

Family origin	Phenotype	Age of onset	Diagnosis	Mutation	Reference
China	auditory neuropathy accompanied with delayed peripheral sensory neuropathy, and hypoplasia of bilateral cochlea nerves showed by MRI	School age & adolescence (average of 12.6 yrs)	AUNX1/DFNX5	11 mutations (pls see table 1)	this study
America	Motor-sensory axonal neuropathy, bilateral sensorineural hearing loss, mental retardation, and abnormal MRI signals in the white matter	childhood (from birth)	Cowchock syndrome	c.1478A>T (p.E493V)	Rinaldi C et al. (2012)
Palestinian	choroids plexus cysts, bilateral brain ventriculomegaly, enlarged cisterna magna, swallowing difficulties, hypotonic with muscle weakness and atrophy	prenatal	Prenatal ventriculomegaly	c.923G>A (p.G308E)	Berger I et al. (2011)
Italy	psychomotor regression, muscle weakness and atrophy, lack of further development, and abnormal MRI signals in the basal ganglia	~1 year of age	COXPD6	c.601–603del (p.R201del)	Ghezzi D et al. (2010)
